# Understanding the Factors That Influence the Antioxidant Activity of Manganosalen Complexes with Neuroprotective Effects

**DOI:** 10.3390/antiox13030265

**Published:** 2024-02-22

**Authors:** Lara Rouco, Rebeca Alvariño, Amparo Alfonso, Sandra Fernández-Fariña, Ana M. González-Noya, Miguel Martínez-Calvo, Rosa Pedrido, Laura Rodríguez-Silva, Marcelino Maneiro

**Affiliations:** 1 Departamento de Química Inorgánica, Facultade de Ciencias, Campus Terra, Universidade de Santiago de Compostela, 27002 Lugo, Spain; lararouco312@gmail.com (L.R.); sandra.fernandez.farina@usc.es (S.F.-F.); laura.rodriguez@usc.es (L.R.-S.); 2 Departamento de Fisiología, Facultade de Veterinaria, IDIS, Universidade de Santiago de Compostela, 27002 Lugo, Spain; 3 Departamento de Farmacología, Facultade de Veterinaria, IDIS, Universidade de Santiago de Compostela, 27002 Lugo, Spain; amparo.alfonso@usc.es; 4 Departamento de Química Inorgánica, Facultade de Química, Universidade de Santiago de Compostela, 15782 Santiago de Compostela, Spain; ana.gonzalez.noya@usc.es (A.M.G.-N.); miguel.martinez.calvo@usc.es (M.M.-C.); rosa.pedrido@usc.es (R.P.)

**Keywords:** reactive oxygen species (ROS), oxidative stress, catalytic antioxidants, superoxide dismutase, catalase, neuroprotection, manganese, Schiff bases

## Abstract

Manganosalen complexes are a class of catalytic antioxidants with beneficial effects against different neurological disorders according to various in vitro and in vivo studies. The interest in the factors that determine their antioxidant activity is based on the fact that they are key to achieving more efficient models. In this work, we report a set of new manganosalen complexes, thoroughly characterized in the solid state and in solution by different techniques. The chelating Schiff base ligands used were prepared from condensation of different substituted hydroxybenzaldehydes with 1,2-diaminoethane and 1,3-diaminopropane. The antioxidant activity of the new models was tested through superoxide dismutase and catalase probes in conjunction with the studies about their neuroprotective effects in human SH-SY5Y neuroblastoma cells in an oxidative stress model. The ability to scavenge excess reactive oxygen species (ROS) varied depending on the manganosalen models, which also yielded different improvements in cell survival. An assessment of the different factors that affect the oxidant activity for these complexes, and others previously reported, revealed the major influence of the structural factors versus the redox properties of the manganosalen complexes.

## 1. Introduction

Manganosalen complexes are a class of synthetic catalysts with antioxidant capacity [1,2,3,4,5]. Their design is inspired by natural enzymes such as catalases, superoxide dismutases, or glutathione peroxidases, which are all part of the body’s antioxidant barrier that controls the excess of reactive oxygen species (ROS) [6,7,8,9,10,11,12,13].

Although the name “manganosalen” derives from the manganese complex with the tetradentate ligand (with ONNO donor atoms), obtained by condensing salicylaldehyde and ethylenediamine (salen), in the current literature, it is extended to all manganese metal complexes derived from the family of tetradentate ligands obtained by reaction between any substituted salicylaldehyde and different diamines. The set of compounds resulting from combining these two variables together with the use of different counterions for the manganese salts is a number that exceeds a couple of thousands by far [14,15,16]. The study of the chemical properties and catalytic activities of this type of compound reveals important differences depending on small variations in the charge donor/acceptor character of the ring substituents or in the nature of the organic structure of the diamine. For example, in the literature [12,17,18,19,20], manganosalen complexes have been described that differ by more than 1000 mV in their redox potential for the Mn(III)/Mn(II) process, with values ranging from −650 mV to +0.480 mV versus a saturated calomel electrode. This is extraordinary for a type of compound that is characterized by mimicking the activity of redox enzymes, such as those mentioned before that are involved in the body’s antioxidant barrier.

The role of these antioxidant enzymes is crucial for controlling the oxidative stress in the brain, an organ with high oxygen consumption (20% of oxygen uptake) and also with a high content of polyunsaturated fatty acids, which are susceptible to oxidative degradation. Oxidative stress can cause different neurodegenerative diseases, such as Alzheimer’s and Parkinson’s diseases, amyotrophic lateral sclerosis, or Huntington’s disease [21,22,23,24,25,26].

Different artificial models of manganosalen complexes with neuroprotective effects have been described. Raber et al. [27] have studied the ability of one of the compounds, labeled EUK-207, to mitigate radiation-induced cognitive damage. Another manganosalen complex, labeled EUK-189 and used by Levine et al. [28], has shown the ability to correct the neurobehavioral abnormality of ataxia-telangiectasia mice. Collins et al. [29] have reported survival prolongation in a mouse model of human prion disease treated with EUK-189. Other studies performed by Melov et al. [30,31] have shown the rescue of mice lacking the mitochondrial form of superoxide dismutase from the oxidative neurodegenerative process using different manganosalen complexes. These compounds have also been reported, by different authors, to show beneficial effects in the prevention of age-dependent cognitive deficits [32,33,34,35,36].

Despite the neuroprotective capacity of manganosalen compounds reported in the literature, there is still a profound lack of knowledge of the structural features that favor antioxidant activity in cellular and/or living models. Since the redox properties, steric effects, or the catalytic mechanism can vary, due to small structural or conformational changes in the molecule, it is necessary to delineate which of the aforementioned characteristics are more appropriate when designing new compounds with better activities in biological media.

This goal constitutes one of the research lines of our group [37,38,39,40]. Our results, based on different catalytic assays, suggest that the antioxidant activity of these types of compounds is carried out through an inner-sphere mechanism, so it is crucial to design complexes that favor this type of catalysis through some labile coordination position where the substrate can bind. Another relevant issue is the importance, decisive or not, of the redox potentials of manganosalen compounds and also the ability to correlate the activity in biological media with the electrochemical behavior of the complexes.

**Scheme 1:**
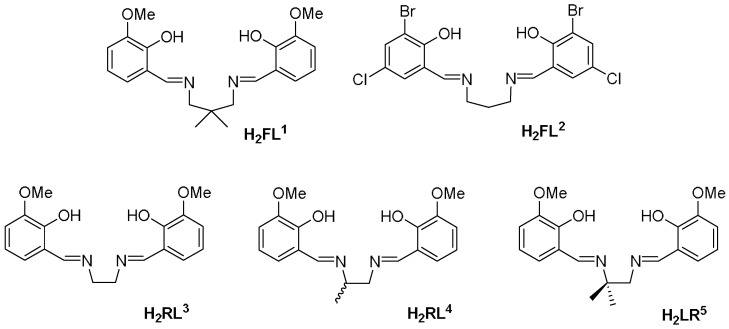
Structure of the flexible ligands (FLs) and rigid ligands (RLs) H_2_FL^1^, H_2_FL^2^,H_2_RL^3^, H_2_RL^4^ and H_2_RL^5^.

This work addresses both issues, in search of a better understanding of the factors that influence the neuroprotective effects of manganosalen complexes. To this end, salen-type ligands with different structural features have been selected, both by modifying the substituents on the aromatic rings and by the greater rigidity (denoted as RL, rigid ligand) or flexibility (denoted as FL, flexible ligand) determined by the length of the methylene chain in the spacer between the imine groups. The donor nature of the electronic charge or the possible participation of the aromatic ring substituents in hydrogen bonding could affect, respectively, either the redox properties of compounds or the establishment of intermolecular interactions. In this sense, two new manganosalen complexes have been prepared and thoroughly characterized (both in solution and in solid state) using the H_2_FL^1^ and H_2_RL^3^ ligands (see Scheme 1 and Scheme S1). The antioxidant activity of these two compounds and another previously reported using the H_2_FL^2^ ligand has been studied using various assays. Their neuroprotective effect against H_2_O_2_-induced oxidative stress in a human neuronal model with SH-SY5Y cells was also examined [41]. Finally, these results of neuroprotection and mitochondrial membrane potential assays have been compared with those previously obtained under comparable conditions, using complexes with ligands H_2_RL^3^, H_2_RL^4^ and H_2_RL^5^ [39]. The role of different ancillary co-ligands, conveniently selected to achieve an increase in the tetragonal elongation in the metal complexes, is also discussed. We expect this structural factor to concomitantly result in an increase in the catalytic activity.

## 2. Materials and Methods

### 2.1. Chemical Synthesis and Characterization

All solvents, 3-methoxy-2-hydroxybenzaldehyde, 3-bromo-5-chloro-2-hydroxybenzaldehyde, 2,2-dimethyl-1,3-diaminepropane, ethylenediamine, 1,3-diaminepropane, manganese(II) perchlorate, manganese(II) acetate tetrahydrate, potassium thiocyanate, tetraethylammonium perchlorate, 4,4′-bipyridine, hydrogen peroxide and tetra butyl hydroperoxide (TBHP) were commercial (Sigma-Aldrich/Merck, Darmstadt, Germany) and used without further purification.

^1^H NMR spectra were recorded at 25 °C on a Bruker Avance 300 MHz spectrometer (Bruker BioSpin, Rheinstetten, Germany). Elemental analyses were performed on a Carlo Erba Model 1108 CHNS-O elemental analyzer (CE Instruments, Wigan, UK). ESI mass spectra were obtained on a Hewlett-Packard model LC-MSD 1100 instrument (positive-ion mode, 98:2 CH_3_OH/HCOOH as the mobile phase, 30–100 V; Hewlett-Packard, Palo Alto, CA, USA). IR spectra were recorded as KBr pellets on a Bio-Rad FTS 135 spectrophotometer (Bio-Rad Laboratories, Hercules, CA, USA). Conductivities of 10^−3^ M solutions in dimethylformamide were obtained on a Crison microCM 2200 conductivity meter (Crison Instruments, Barcelona, Spain). Room-temperature magnetic moments were measured using an MSB-MKI system (Sherwood Scientific, Cambridge, UK). Electronic spectra were registered on a Cary 50 spectrometer (Agilent Technologies, Stockport, UK). EPR measurements were recorded on a Bruker ESP300E X-band spectrometer equipped with an ER4116DM dual-mode cavity (Bruker Physik AG, Karlsruhe, Germany). The Chem3D 15.1 MM2 program (PerkinElmer, Waltham, MA, USA) was used for structure calculations.

Electrochemical measurements were performed using an Autolab PGSTAT101 potentiostat (Metrohm Autolab, Utrecht, Netherlands) working with a three-electrode configuration. The working electrode was a glassy carbon disc (Metrohm 6.1204.300). A Ag/AgCl reference electrode filled with 3 M KCl (Metrohm 6.0728.000) was used as the reference electrode, while a Pt wire was used as the counter electrode. Measurements were made with ca. 2 × 10^−3^ M solutions of complexes in DMSO containing 0.1 M tetraethylammonium perchlorate as a supporting electrolyte. The solutions were deoxygenated before each measurement by bubbling N_2_. The graphite disc working electrode was polished before each experiment using a polishing kit (Metrohm 6.2802.010), first with α-Al_2_O_3_ (0.3 μm) and then washed with distilled water. Salen-type ligands FL^1^, FL^2^ and RL^3^ were prepared according to the literature [42] by condensation of the substituted 2-hydroxybenzaldehyde and the corresponding diamine in chloroform, and their synthesis and characterization have been already reported [37]. Complex **2** has been prepared as previously reported [43].

[MnFL^1^(NCS)(CH_3_CH_2_OH)]·H_2_O (**1**): The ligand H_2_FL^1^ (0.20 g, 0.54 mmol) was dissolved in 30 mL of ethanol, and the resulting yellow solution was stirred. A solution of manganese(II) acetate tetrahydrate (0.13 g, 0.54 mmol) in ethanol (25 mL) was added to the stirred solution, which changed its color to brown. The progress of the reaction under air was followed by TLC. After 2 h of stirring with gentle heating, 0.04 g (0.54 mmol) of KSCN in 10 mL of methanol was added. Then, the solution was stirred with gentle heating for 1 h before concentration by slow evaporation. The compound was obtained as brown crystals, which were isolated by filtration, washed with diethyl ether and dried in air. Yield 70%. Anal. Calc. for C_24_H_32_MnN_3_O_6_S (545.53): C, 52.84; H, 5.91; N, 7.70; S, 5.88. Found: C, 52.10; H, 5.68; N, 7.73; S, 5.94 %. MS ES (m/z) 423 [MnFL^1^]^+^; IR (KBr, cm^−1^): ν(O-H) 3427, ν(C=N) 1614, ν(C-O) 1252, ν(SCN) 2073, ν(C-S) 733; ^1^H NMR (DMSO-d_6_, ppm): δ −20.8 (H4), −15.8 (H5). *μ* = 4.8 *μ*_B_. *Λ*_M_ = 46 μS.

[(MnRL^3^)_2_(4,4-bipy)(H_2_O)_2_].2(CH_3_COO)·12H_2_O (**3**): To a methanolic solution of 0.20 g (0.60 mmol) of H_2_RL^3^, 0.15 g (0.60 mmol) of Mn(CH_3_COO)_2_ was added, and the color of the stirred solution changed from yellow to brown. The progress of the reaction under air was followed by TLC. After 2 h of stirring with gentle heating, 0.09 (0.60 mmol) of 4,4-bypiridine in 15 mL of methanol was added. The reaction was carried out for 1 h under stirring with gentle heating. The resulting brownish solution was first concentrated under reduced pressure until half of its volume and then left to crystallize by slow evaporation. The brown crystals formed were filtered and washed with diethyl ether. Yield 40%. Anal. Calc. for C_50_H_78_Mn_2_N_6_O_26_ (1289.06): C, 46.59; H, 6.10; N, 6.52. Found: C, 46.9; H, 6.11; N, 6.48%. MS ES (m/z) 381.3 [MnRL^3^]^+^; IR (KBr, cm^−1^): ν(O-H) 3406, ν(C=N) 1616, ν(C-O) 1256, ν_as_(COO^−^) 1574, ν_s_(COO^−^) 1404; ^1^H NMR (DMSO-d_6_, ppm): δ −20.75 (H4), −16.23 (H5). *μ* = 4.8 *μ*_B_. *Λ*_M_ = 117.3 μS.

### 2.2. Studies of the Catalase and Superoxide Dismutase Activity

The catalase function of the manganosalen complexes was measured by a volumetric test. During the experiment, a methanolic solution of the complex (3 mL, 1 mM) was placed into a 10 mL container, sealed with a septum and connected through a double-ended needle to a gas measuring burette (precision of 0.1 mL). The evolved oxygen was volumetrically measured after the injection, using a syringe, of a H_2_O_2_ solution (1 mL, 2.5 M) through the septum to the thermostatted complex solution under stirring.

The SOD activity was evaluated through the compound’s ability to compete with ferricytochrome *c* for the superoxide radical anion generated by the xanthine/xanthine oxidase (XO) system. XO (from bovine milk), xanthine, ferricytochrome *c* (horse heart) and methanol were from Sigma; phosphate salts were from Scharlau (di-sodium hydrogen phosphate dihydrate and sodium dihydrogen phosphate anhydrous). The reduction of cytochrome *c* was followed at 550 nm. The reaction mixture contained 50 mM phosphate buffer, pH 7.3, 50 mM xanthine and 10 mM ferricytochrome *c*, in a final volume of 1 mL. Sufficient purified XO was added to give a D_Abs(550nm)_/_min_ of 0.025 (this corresponds to a superoxide formation rate of 1.2 mM/min (D_e_ = 21,000 M^−1^ cm^−1^)). After defining the suitable volume of XO, different concentrations of each compound were tested in order to determine the one that inhibits the rate of cytochrome *c* reduction by 50%. Each compound was first dissolved in methanol and then diluted in water. Each rate determination (without and with each compound) was repeated at least three times.

### 2.3. Cell Culture

In this study, a human neuroblastoma SH-SY5Y cell line was used, previously purchased from the American Type Culture Collection (ATCC), number CRL2266. Cells were grown in Dulbecco`s modified Eagle’s medium: Nutrient Mix F-12 (DMEM/F-12) supplemented with 10% fetal bovine serum (FBS), 100 U/mL penicillin, glutamax (1%) and 100 μg/mL streptomycin. Cells were incubated in a humidified atmosphere of 5% CO_2_ at 37 °C and dissociated weekly using 0.05% trypsin/EDTA. All reagents were provided by Thermo Fisher Scientific (Waltham, MA, USA). For cell treatment, compounds were dissolved in DMSO, and serial dilutions were performed in cell medium. DMSO concentration was kept under 0.5% in all cases.

### 2.4. Cell Viability Assay 

SH-SY5Y cells were seeded, 24 h before the experiments were carried out, in 96-well plates at a density of 5 × 10^4^ cells per well. Human neuroblastoma cells were treated with manganosalen complexes **1**–**3** at different concentrations (0.001, 0.01, 0.1, 1 and 10 μM) for 24 h, and the MTT (3-(4, 5-dimethyl thiazol-2-yl)-2,5-diphenyl tetrazolium bromide) assay was used to evaluate the effect of complexes on cell viability [44,45]. After treatment, cells were rinsed three times with Locke’s buffer. SH-SY5Y cells were incubated for 1 h at 37 °C with 500 μM of MTT (Merck, Darmstadt, Germany) dissolved in saline buffer and then disaggregated with 5% sodium dodecyl sulfate. A spectrometer plate reader was used to measure the absorbance of formazan crystals at 595 nm. Saponin from Quillaja bark (Merck) was used as cellular death control, and its absorbance was subtracted from the other values.

### 2.5. Neuroprotection and Mitochondrial Membrane Potential Assays

For both assays, SH-SY5Y cells were seeded in 96-well plates at a density of 5 × 10^4^ cells per well, 24 h before the experiments were carried out, and then treated with **1**–**3** at different concentrations (0.001, 0.01, 0.1 and 1 μM) and 75 μM TBHP for 6 h. An MTT probe was used to evaluate the ability of manganosalen complexes to protect cells from TBHP damage. The assay was carried out as described above. A TMRM (tetramethyl rhodamine methyl ester) probe was employed to assess the mitochondrial membrane potential. After incubation, Locke’s buffer was added to each well, and the cells were rinsed two times. Then, SH-SY5Y cells were incubated at 37 °C for 30 min with 1 μM TMRM. Next, cells were solubilized with DMSO and H_2_O at 50%, and the fluorescence was monitored with a spectrophotometer plate reader (535 nm excitation and 590 nm emission). All experiments were performed at least three times in triplicate. The endogenous antioxidant vitamin E (vitE) at 25 μM was used as a positive control to validate the in vitro model in all the assays. Data are presented as mean ± SEM. Statistical differences were evaluated by Student’s *t* tests with Graph Pad Prism 6 software. Statistical significance was considered at *p* < 0.05.

### 2.6. Crystallographic Studies

Single crystals of **1** and **3**, suitable for X-ray diffraction studies, were obtained by slow evaporation as detailed in the synthetic section. Both structures were collected on a Bruker Smart CCD 1000 diffractometer at low temperatures (100–110 K) using graphite-monochromated *Mo-Kα* radiation (λ = 0.71069 Å). Crystal data collection and refinement are summarized in Table S1. The two structures were solved by direct methods [46] and finally refined by full-matrix least squares based on F^2^. SADABS [47] was used to apply an empirical absorption correction. F^2^ was refined against ALL reflections. The weighted R-factor R_w_(F^2^) is based on F^2^, and conventional R-factors R are based on F, with F set to zero for negative F^2^. The threshold expression of F^2^ > 2σ(F^2^) is used only for calculating R-factors(gt), for example, and is not relevant to the choice of reflections for refinement. R-factors based on F^2^ are statistically about twice as large as those based on F. Molecular graphics were created with ORTEP [48] and Mercury [49].

## 3. Results

### 3.1. Synthesis and Characterization of the Manganosalen Complexes

Manganosalen complexes **1**–**3** were obtained in high yield as outlined in the experimental section. Characterization of complexes **2** and **4**–**6** has been already reported [39,43], so the results of this subsection will be focused on complexes **1** and **3**. The crystalline nature of compounds **1** and **3** allows them to be obtained with a high degree of purity. Both complexes were characterized in solid state (microanalysis, IR spectroscopy, magnetic moments and X-ray diffraction studies) and in solution (UV and ^1^H NMR spectroscopies, EPR spectroscopy, conductivity and cyclic voltammetry behavior).

The combination of all these techniques allows for establishing the formula and structure of the two compounds. In both cases, manganese is found as Mn(III) with magnetic moment values of 4.8 *μ*_B_, very close to expected for magnetically diluted high-spin d^4^ ions, which is corroborated in the spectra obtained by paramagnetic ^1^H NMR spectroscopy (Figure S1), with two high-field resonances due to the isotropic shift of the protons of the salen ligand in high-spin Mn(III) complexes in an octahedral field [37,38]. UV-Vis spectra (Figure S2) show bands at about 530–600 nm (ε = 670–800 L mol^−1^ cm^−1^) attributable to ^5^E_g_ → ^5^T_2g_ transitions for a Jahn–Teller-distorted d^4^ manganese ion [50]. Stability studies over time of solutions of **1** and **3** have been carried out. UV-Vis confirms the stability of the complexes during the incubation time periods used in the biological studies.

Elemental analysis and conductivity measurements are consistent with the proposed formulas, which imply a non-electrolyte behavior for **1** and a 1:1 (2:2) electrolyte for **3** [51]. The coordination of the Schiff ligands (H_2_FL^1^ in **1** and H_2_RL^3^ in **3**) to the manganese is verified by IR spectroscopy and ESI mass spectrometry (Figures S3 and S4). In both complexes, the appearance of the C=N and C-O bands of the Schiff base is observed in the IR spectra and their shift to lower frequencies in relation to the free ligand. In the case of complex **1**, the ν(SCN) and ν(C-S) corresponding to the coordinated thiocyanate group through the N atom are also observed [52].

X-ray crystallographic studies allow the precise structural characterization of complexes **1** and **3** in the solid state. In both cases, the crystalline structures show the coordination of the ligand (H_2_LF^1^ and H_2_RF^3^, respectively) to the manganese ion through the imine nitrogen atoms and the phenolic oxygen atoms of the Schiff base. Selected bond distances and angles for both structures are given in Tables S2 and S3 of the Supplementary Material. Confirming the data obtained by other characterization techniques, the Mn(III) ion is arranged in a distorted octahedral environment, with the salen-type ligand accommodated in the equatorial plane and the axial positions occupied by other molecules.

**Figure 1:**
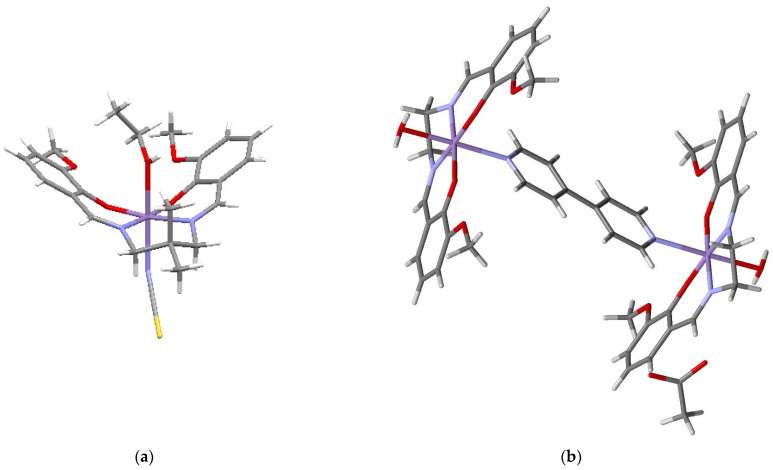
Crystal structures for **1** and **3**: (**a**) Stick diagram for **1**, showing the coordination of the bideprotonated Schiff base ligand LF^1^, the thiocyanate and an ethanol molecule to the manganese ion; (**b**) stick diagram for **3** showing the 4,4′-bipyridine bridge between two manganese–salen moieties with bideprotonated RL^3^ ligand; solvate ions are omitted for clarity (manganese ions in purple; nitrogen in blue; oxygen in red; carbon in grey; hydrogen in white).

Figure 1 shows the crystal structures of **1** and **3**. In the case of **1**, the axial positions are occupied by a thiocyanate group bonded to the metal ion through the nitrogen atom, and by a molecule of ethanol, which is bound to manganese through the oxygen atom. The dianionic FL^1^ ligand is displaced in a non-planar arrangement, bent towards the ethanol ligand and away from the thiocyanate (the dihedral angle between the aromatic rings is 66.74°, see Figures S5 and S6). The bond distances in the axial positions are larger than the equatorial ones, showing the typical Jahn–Teller effect for Mn(III) ions [37,38,39]. For example, the axial Mn-O(19) bond distance is 2.2969(13) Å, while the equatorial Mn-O(13A) and Mn-O(13B) bond distances are between 1.8844(12) and 1.8941(12) Å. The coordination of the thiocyanate ion is nearly linear, the Mn-N-C angle being 174.91(5)°. A solvent water molecule is present in the lattice, which is hydrogen bonded to the complex molecule to build a one-dimensional chain (Figure S7).

Complex **3** is a dimer containing two units [Mn(RL^3^)(H_2_O)]. OAc.6H_2_O is bridged by a 4,4′-bipyridine molecule. In this case, the distorted octahedral environment of the metal ion is completed with the binding of a molecule of water through the oxygen atom and a nitrogen atom of the 4,4′-bipyridine. The tetragonal elongation of the structure is clear since the bond distances of these axial positions are again greater than those of the equatorial positions. Thus, the axial Mn-N distance is 2.3296(19) Å versus equatorial Mn-N distances ranging from 1.2695(19) to 1.9800(19) Å, whereas the axial Mn-O distance is 2.3072(17) Å versus equatorial Mn-O distances ranging from 1.8779(15) to 1.8819(16) Å.

The axial water molecules are implied in a hydrogen bonding scheme with the neighboring dimer (Table S4), causing the approach of manganese atoms of two neighboring molecules to a distance as short as 4.665 Å (Figure S8). The H(31A) of the O(31) of the coordinated H_2_O binds to the phenolic O(9) and the methoxy O(2) of a neighboring molecule. In addition, the H(31B) of the O(31) of the coordinated H_2_O binds to the phenolic O(22) and the methoxy O(23) of the neighboring molecule. From a supramolecular perspective, chains are formed through hydrogen bonds between the occluded water molecules. The molecule is thus an aggregate generated by the three-dimensional growth of the chains formed through hydrogen bonding (Figure S9).

Parallel-mode EPR spectroscopy studies allow a more accurate characterization of the complexes in solution. In this case, the results are consistent with those obtained in the solid state. The Mn(III) ion, a d^4^ system, has an integer electron spin S = 2, and its paramagnetic resonance spectrum can be well elucidated using the microwave field B_1_ parallel to the static field B_0_ [53] with a characteristic six-line pattern.

Figure S10 shows the EPR spectra for **1** and **3** in frozen toluene–dimethylformamide–ethanol (2:1–drop) solutions, recorded at 9 K. The Y axis indicates EPR intensity using an arbitrary scale to highlight the difference between both spectra.

Manganosalen complexes **1** and **3** exhibit the sextet centered at g_eff_ = 8.09, but they are distinguished by their hyperfine coupling due to a ^55^Mn nuclear spin (I = 5/2), A_‖_ = 55 for **1** and A_‖_ = 43 for **3**. The higher value of the hyperfine coupling constant is related to a decrease in the covalence of the N_2_O_2_-chelating atoms of the salen-type ligand, which could be attributed to longer Mn-O and Mn-N distances in **1** compared to **3** [54]. From the structural point of view, longer A_‖_ also implies higher symmetric octahedral coordination while shorter A_‖_ means a greater tetragonal distortion of the structure in the solution state [55,56].

Figure S11 presents the cyclic voltammograms for **1** and **3,** showing the reduction and oxidation waves at different scan rates. The electrochemical data are summarized in Table 1. Both manganosalen complexes display a quasi-reversible Mn(III)/Mn(II) electrochemical behavior. Complex **1** undergoes one reduction process, Mn(III) → Mn(II) at −24 mV, while the reduction of **2** takes place at −10 mV. Peak-to-peak separation (ΔEp = E_pa_ − E_pc_) is 83 mV for **1** and 126 mV for **3** at a 0.02 V s^−1^ scan rate. The reversible character decreased as scan rates increased. Cyclic voltammograms of **1** and **3** support the purity and stability of both complexes in solution. The peak currents of the anodic and cathodic waves (Figures S12 and S13) show linear dependence with the square root of the scan rate, suggesting a diffusion-controlled process.

### 3.2. Catalase and Superoxide Dismutase Activities

The catalase-like function of the compounds has been measured by volumetric determination of the evolved O_2_ after catalytic disproportionation of hydrogen peroxide, which was used in a large excess (>830). Table 1 presents the results in terms of the percentage of decomposed H_2_O_2_ (conversion) and the turnover number (TON) calculated as the number of moles of hydrogen peroxide that a mole of catalyst can decompose before becoming inactivated. Table 1 also includes the values of antioxidant activities of complexes **4**–**6**, previously reported [39]. Two trends of catalase activity can be clearly identified. Complexes **1** and **2**, with a salen-type flexible ligand, do not show significant catalytic activities, whereas complex **3** achieves turnover numbers of about 150; complexes **4**–**6** exhibit TON values of 192–225. Experimental conditions were chosen to compare the results with other previous studies, although the TON values can be increased using different concentration ratios between the substrate and the manganosalen complexes.

Superoxide dismutation activity for **1**–**3** was measured based on the method described by McCord and Fridovich for true SOD enzymes [44], in which the xanthine/XO system is the source of superoxide and cytochrome *c* is used to quantify the superoxide formation. If a compound reacts with superoxide, the reduction of cytochrome *c* in its presence will be decreased, and this inhibition can be used as a measure of the SOD activity of the compound. In practice, one unit of SOD activity is defined as the compound concentration which gives 50% inhibition of the rate of reduction of cytochrome *c*. For each compound (at the concentration that gives 50% inhibition of the rate of cytochrome *c* reduction), it was confirmed that XO is not inhibited. This was achieved by comparing the urate formation, at 295 nm, in the absence and in the presence of the compound. Complexes **1**–**3** interact with superoxide ions, catalyzing their dismutation to molecular oxygen and hydrogen peroxide. The results are listed in Table 1. Complex **3** shows SOD rates close to the equivalent of one SOD unit, a value two times better than that obtained for complexes **1**–**2**.

### 3.3. Cytotoxicity, Neuroprotection and Mitochondrial Membrane Potential Results for Manganosalen Complexes ***1**–**3***

**Figure 2:**
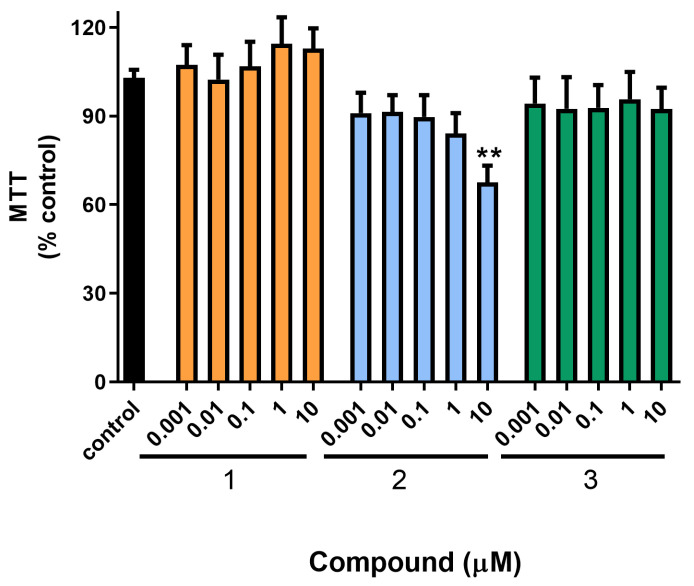
Cell viability of SH-SY5Y cells treated with **1**–**3**. Compounds were added to cells for 24 h, and their cytotoxic effects were determined with MTT assay. Values are mean ± SEM of three independent replicates performed in triplicate, expressed as percentage of untreated control cells. Statistical differences were determined by Student´s *t* test. ** *p* < 0.01.

SH-SY5Y human neuroblastoma cells were treated with different concentrations (0.001, 0.01, 0.1, 1 and 10 μM) of manganosalen complexes **1**–**3**. Figure 2 shows the cytotoxic effects of the complexes, determined with the MTT assay. Cell viability is reduced by complex **2**, particularly at the highest concentrations (10 μM), while compounds **1**–**3** did not present cytotoxic effects at any concentration.

**Figure 3:**
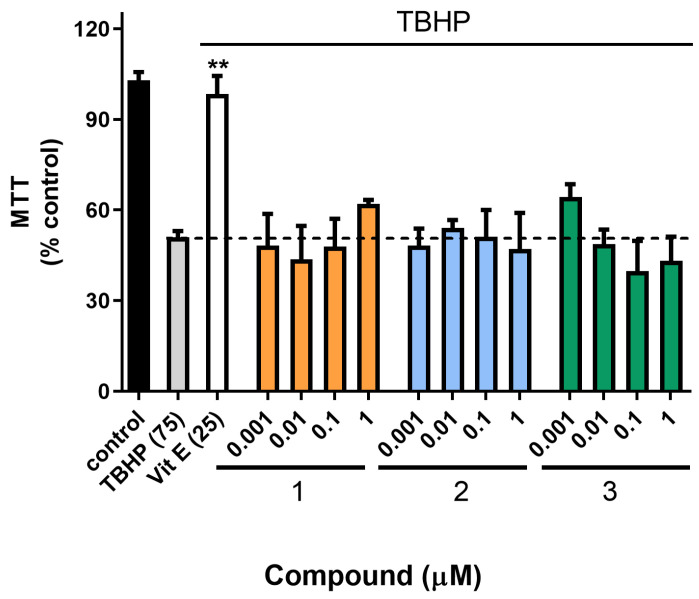
Neuroprotective effect of **1**–**3** in an oxidative stress model. Human neuroblastoma cells were co-treated with 75 μM TBHP and complexes (0.001–1 μM) for 6 h. MTT test was used to assess cell viability. Data are mean ± SEM of three independent replicates. Values are expressed as percentage of control cells and compared to cells treated with TBHP alone by Student’s *t* test. ** *p* < 0.01.

For the oxidative stress studies, the oxidant TBHP was used instead of H_2_O_2_ due to its higher stability in physiological media [57]. In order to determine the neuroprotective effects of **1**–**3**, cells were co-treated with complexes at non-toxic concentrations (0.001, 0.01, 0.1 and 1 μM) and 0.75 μM of TBHP for 6 h. The results are shown in Figure 3.

The addition of TBHP reduced cell viability to 51.0 ± 2.0% (*p* < 0.001). Treatment with **3** produced a slight increase in cell survival at 0.001 μM (64.3 ± 4.7%), although not statistically significant. The protective effects in this case are not dose-dependent. This behavior was already found for other compounds showing antioxidant activity at low concentrations but producing oxidative stress at high concentrations [58,59].

**Figure 4:**
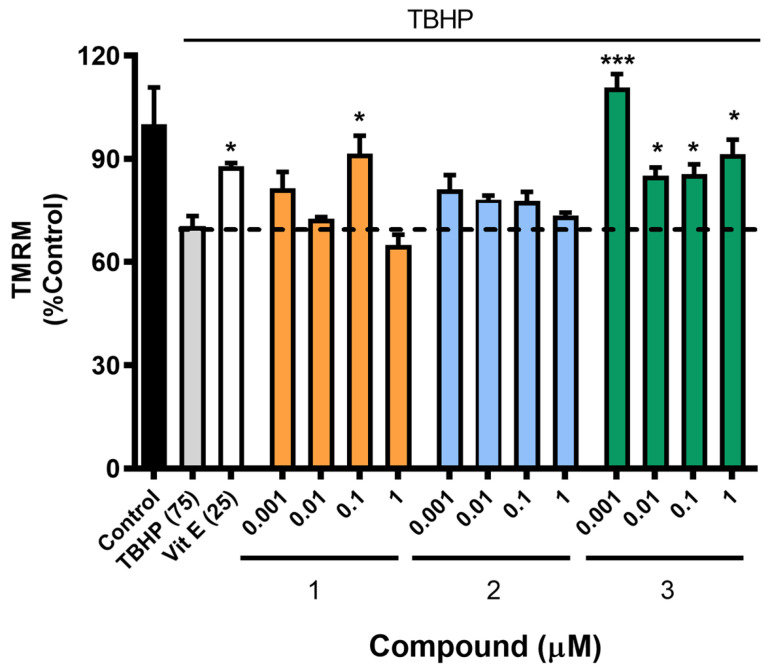
Effect of manganosalen complexes on ΔΨm in SH-SY5Y cells, treated with **1**–**3** (0.001, 0.01, 0.1 and 1 μM) and 75 μM TBHP for 6 h. TMRM dye was employed to evaluate their effect on the mitochondrial membrane potential. Data are mean ± SEM of three independent replicates. Values are expressed as percentage of control cells and compared to cells treated with TBHP alone (* *p* < 0.05, *** *p* < 0.001) by Student’s *t* test.

Then, the potential of the manganosalen complexes for restoration of mitochondrial function in this human neuronal model was studied with TMRM dye. While compounds **1** and **2** did not show an effect on the restoration of mitochondrial membrane potential (ΔΨm), manganosalen complex **3** produced an augmentation at all concentrations (Figure 4).

## 4. Discussion

One of the crucial issues for the efficacy of synthetic complexes that mimic the antioxidant enzymes of the organism is their redox behavior, since the processes that they have to catalyze involve oxidation/reduction reactions [60]. To this end, it is essential that the complexes can be easily oxidized and reduced, which in terms of electrochemical behavior we describe as the ability to display reversible or quasi-reversible redox processes. The potential redox value of the compound is also important. Its proximity to zero volts allows redox processes to occur more easily in biological media [17,18,19,20].

Manganosalen compounds generally meet both requirements and therefore can be considered functional models of various redox enzymes. The compounds described in this work have potentials close to zero volts, and their oxidation and reduction waves show a high degree of reversibility. However, while these redox conditions are necessary to obtain good synthetic antioxidant catalysts, we have found in this work that redox behavior is not the only variable involved. In fact, it is not the key factor for improving the antioxidant activity and the neuroprotective effects in the human neuronal model used.

**Figure 5:**
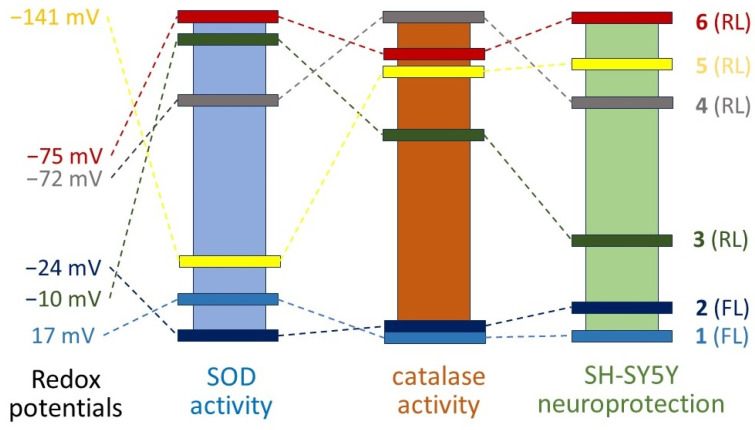
Qualitative comparison between the redox potentials (E_1/2_) and the SOD, catalase and neuroprotective effects for manganese compounds **1**–**6**. FL denotes flexible salen-type ligand while RL means rigid ligand. Each column shows the order of activities between the compounds, although not in a strictly quantitative way.

The results show that not all manganosalen compounds are active in antioxidant assays or protect cells against oxidative damage, and this difference in activity is not correlated with their redox behavior (Figure 5) but is conditioned by other structural factors. In addition, the results show some consistency between the antioxidant assays, such as the simple catalase assay performed, and the biological studies. This is especially relevant, as it could allow a simpler experimental design to be implemented in the future by pursuing a correct choice or discounting less optimal synthetic models for biological assays.

Without a doubt, a relevant issue is whether the mechanism of the antioxidant activity for this type of compound is carried out through the inner-sphere or the outer-sphere modes [18,19,61]. In the inner-sphere mechanism, the substrate molecule binds to the metal ion, entering into what is known as the first coordination sphere of the metal complex. For this to happen, the complex must have a vacancy in this coordination sphere or a labile ligand that can be easily replaced by the substrate. Our previous experience and that of other research groups suggest that this is the mechanism that governs the antioxidant catalysis of manganosalen complexes [15,16,17,18,19,37,38,39,40].

The ability to bind the substrate molecule for subsequent oxidation will depend on structural issues. In the present study, we found two clearly differentiated behaviors regarding the antioxidant and neuroprotective activity of the compounds. Complexes **1**–**2** present lower antioxidant capacities in all the conducted assays, with both having in common a longer spacer chain between the imine nitrogen atoms. The different characterization techniques used and the theoretical modeling studies performed for the complexes agree that the final structure of the metal complexes is conditioned by the length of this aliphatic chain. Figures S14 and S15 show the representations of complexes **1**–**6** obtained by MM2 calculations, which are in accordance with the structures obtained by X-ray diffraction studies.

The limited ability to coordinate a substrate molecule exhibited by **1**–**2** can be explained by a conjunction of structural factors: (i) the aromatic rings of the flexible ligands are bent in **1**–**2**, forming a dihedral angle of approximately 67° (see Figures S6 and S14), which creates steric hindrance for the approach of a substrate molecule; (ii) complexes **1**–**2** with flexible ligands display octahedral environments around the metal ion with greater symmetry than **3**–**6** with rigid ligands and experience the pincer effect because of the shorter distances between the imine nitrogen atoms (Figure S15). As a result of this effect, the octahedral coordination environment around the metal ion is more distorted, thus increasing the lability of one of the apical positions [37].

EPR results also support a greater tetragonal distortion of the structure for **3**–**6** [55,56]. This tetragonal distortion makes one of the apical positions more labile, therefore facilitating the appearance of a vacancy where the substrate molecule would subsequently be placed.

The ancillary ligands used in our research in recent years (dicyanamide, thiocyanate, 4,4′-bipyridine) have also been selected to achieve an increase in this tetragonal distortion. The obtained results show that they are effective for achieving this type of structure. However, further research is needed on other effects (solubility, toxicity) that these co-ligands can produce. For example, in the case of compound **3**, it is found that the co-ligand acts as a bridge between the metal ions, blocking those coordination positions, which could explain its lower activity with respect to **4**–**6**.

Complexes **3**–**6** show better antioxidant behavior in the three types of studies performed. Figure 5 illustrates the idea of this discussion with a qualitative comparison between the redox behavior, antioxidant activity, and the type of ligand, flexible or rigid, present in each compound. There are other additional factors that can modulate the neuroprotective effects of manganese–salen complexes, such as their previous toxicity, solubility, lipophilicity and possible hormetic effect. For example, the different activities of **3**–**6**, which may be affected by one or several of these factors, can be checked. However, it is also true that we are advancing in obtaining the right set of tools that will allow us to discard a priori a series of models and design other new, more efficient ones.

## 5. Conclusions

Manganosalen complexes show neuroprotective effects in a human neuronal model. Their antioxidant activity is derived, in part, from their ability to catalyze redox processes in a reversible or quasi-reversible manner. In this study, it has been found that not all complexes are actually active, and this fact can be correlated with structural factors. The precise characterization of the compounds in the solid state and in solution has allowed establishing that those with the ability to accommodate the substrate molecule are more active, both in antioxidant and cellular neuroprotection assays. Manganosalen complexes with shorter spacers between the imine groups give rise to more rigid ligands, which facilitate environments with a greater tetragonal elongation. These environments, which can be conveniently characterized by the EPR spectroscopy technique, are the ones that allow vacancies or contain labile ligands that can be replaced by the substrate to be oxidized. This type of compound shows the best activity results in all types of assays performed, which is consistent with an inner-sphere mechanism.

## Figures and Tables

**Table 1:** Redox potentials (vs Ag/AgCl saturated) at 0.02 V s^−1^ scan rate, SOD and catalase activities for **1**–**6**.

Complex	E_1/2_ (mV) ^a^	ΔEp ^b^	Catalase Activity ^c^		SOD Activity EC50 (μM) ^d^
			Conversion	TON	
**1** (FL) ^e^	−24	83	2 ± 1	15 ± 8	1.95
**2** (FL)	17	212	3 ± 1	24 ± 8	2.00
**3** (RL)	−10	126	18 ± 2	150 ± 17	0.92
**4** (RL)	−72	110	27 ± 3	225 ± 25	1.20
**5** (RL)	−141	111	23 ± 2	192 ± 17	1.90
**6** (RL)	−75	196	24 ± 2	200 ± 17	0.85

^a^ Half-wave potential; ^b^ peak-to-peak separation; ^c^ catalase activity expressed as percentage of H_2_O_2_ decomposed after 60 min (conversion) and turnover number (TON); ^d^ SOD activity expressed as half-maximal effective concentration (EC_50_) values; ^e^ FL denotes flexible ligand and RL rigid ligand.

## Data Availability

Crystallographic data for **1** and **3** were deposited into the Cambridge Crystallographic Data Centre, CCDC 2310803 and 2310826. These data can be obtained via www.ccdc.cam.ac.uk/data_request/cif (31 January 2024), by emailing data_request@ccdc.cam.ac.uk or by contacting The Cambridge Crystallographic Data Centre, 12 Union Road, Cambridge CB2 1EZ, UK; Fax: +44-1223-336033.

## Supplementary Materials

The following supporting information can be downloaded at https://www.mdpi.com/article/10.3390/antiox13030265/s1: Table S1: Crystal data and refinement of **1** and **3**; Table S2: Selected bond lengths (Å) and angles (°) for **1**; Table S3: Selected bond lengths (Å) and angles (°) for **3**; Table S4: Hydrogen bond scheme (distances and angles) for **3**; Scheme S1: Scheme of the Schiff base ligands H_2_FL^1^, H_2_FL^2^, H_2_RL^3^, H_2_RL^4^ and H_2_RL^5^ and their reactions resulting in complexes **1**–**6**; Figure S1: Paramagnetic ^1^H NMR spectra for **1** (a) and **3** (b); Figure S2: UV-Vis spectra for **1** (a) and **3** (b); Figure S3: IR spectra for **1** (a) and **3** (b); Figure S4: ESI mass spectra for **1** (a) and **3** (b); Figure S5: ORTEP view and atom numbering scheme for **1**; Figure S6: Representation of the dihedral angle (value of 66.74°) between the aromatic rings of the salen-type ligand in **1**; Figure S7: Stick diagram of **1** showing the supramolecular 1D chain through hydrogen bonding; Figure S8: Hydrogen bonding between axial water molecules and imine N atom and phenolic O atom from the neighboring dimeric complex in **3** causes an intermolecular Mn…Mn distance of 4.665 Å, shorter than the 11.710 Å for the Mn…Mn distance inside the dimer; Figure S9: Three-dimensional a-axis view for **3**; Figure S10: Parallel-mode EPR spectra for **1** and **3**; Figure S11: Cyclic voltammograms for **1** and **3** at different scan rates; Figure S12: Plot of the linear dependence of anodic and cathodic peak currents with the square root of the scan rate of **1**; Figure S13: Plot of the linear dependence of anodic and cathodic peak currents with the square root of the scan rate of **3**; Figure S14: Simulation stick diagrams for **1**–**6** obtained by MM2 calculations; Figure S15: Stick diagram views obtained by MM2 calculations for **1**–**6** showing the equatorial manganese coordination core for the complexes. Checkcifs for **1** and **3**.
