# Supplementary material for: Understanding the Factors That Influence the Antioxidant Activity of Manganosalen Complexes with Neuroprotective Effects
Source: Antioxidants (Basel). 2024 Feb 22;13(3):265. doi: 10.3390/antiox13030265 (PMC10967637; doi:10.3390/antiox13030265)

Supplementary Materials for

Understanding the factors that influence the antioxidant activity of manganosalen complexes with neuroprotective effects

Lara Rouco[1], Rebeca Alvariño[2,*], Amparo Alfonso[3], Sandra Fernández-Fariña[1], Ana M. González-Noya[4], Miguel Martínez-Calvo[4], Rosa Pedrido[4], Laura Rodríguez-Silva[1] and Marcelino Maneiro[1,*]

[1] Departamento de Química Inorgánica, Facultade de Ciencias, Campus Terra, Universidade de Santiago de Compostela, 27002 Lugo, Spain; lara.rouco.mendez@usc.es; sandra.fernandez.farina@usc.es; marcelino.maneiro@usc.es; laura.rodriguez@usc.es

[2] Departamento de Fisiología, Facultade de Veterinaria, IDIS, Universidade de Santiago de Compostela, 27002 Lugo, Spain; rebeca.alvarino@usc.es

[3] Departamento de Farmacología, Facultade de Veterinaria, IDIS, Universidade de Santiago de Compostela, 27002 Lugo, Spain; amparo.alfonso@usc.es

[4] Departamento de Química Inorgánica, Facultade de Química, Universidade de Santiago de Compostela, 15782 Santiago de Compostela, Spain; ana.gonzalez.noya@usc.es; miguel.martinez.calvo@usc.es; rosa.pedrido@usc.es

* Correspondence: rebeca.alvarino@usc.es; marcelino.maneiro@usc.es; Tel.: +34 982 824106

Table of contents

Table S1. Crystal data and refinement for **1** and **3**

Table S2. Selected bond lengths (Å) and angles (º) for **1**

Table S3. Selected bond lengths (Å) and angles (º) for **3**

Table S4. Hydrogen bond scheme (distances and angles) for **3**

Scheme S1. Scheme of the Schiff base ligands H_2FL^1, H_2FL^2, H_2RL^3, H_2RL^4 and H_2RL^5, and their reactions to obtain complexes **1**-**6**.

Figure S1. Paramagnetic ^1H NMR spectra for **1** (a) and **3** (b)

Figure S2. UV-vis spectra for **1** (a) and **3** (b)

Figure S3. IR spectra for **1**(a) and **3** (b)

Figure S4. ESI mass spectra for **1** (a) and **3** (b)

Figure S5. ORTEP view and atom numbering scheme for **1**, showing the coordination of the Schiff base ligand FL^1, the thiocyanate and etanol molecule to the manganese ion; hydrogen atoms are omitted for clarity.

Figure S6. Representation of the dihedral angle (value of 66.74º) between the aromatic rings of the salen-type ligand in **1**

Figure S7. Stick diagram of **1** showing the supramolecular 1D chain through hydrogen bonding

Figure S8. Hydrogen bonding between axial water molecules and iminic N atom and phenolic O atom from de neighboring dimeric complex in **3** causes intermolecular a Mn…Mn distance of 4.665 Å, shorter than the 11.710 Å for the Mn…Mn distance inside de dimer

Figure S9. Three-dimensional a-axis view for **3**

Figure S10. Parallel-mode electron paramagnetic resonance (EPR) spectra for **1** and **3** in frozen toluene:dimethylformamide:ethanol (2:1:drop) solutions. EPR conditions: microwave frequency, 9.37 GHz; temperature, 9 K; microwave power, 2 mW.

Figure S11. Cyclic voltammograms for **1** and **3** at different scan rates: 0.02 V s^{-1} (blue), 0.05 V s^{-1} (green) and 0.09 V s^{-1} (red).

Figure S12. Plot of the linear dependence of anodic and cathodic peak currents with the square root of the scan rate of **1**.

Figure S13. Plot of the linear dependence of anodic and cathodic peak currents with the square root of the scan rate of **3**.

Figure S14. Simulation stick diagrams for **1**-**6** obtained by MM2 calculations to get the minimum energy perspective plots for the geometry of the complexes

Figure S15. Stick diagram views obtained by MM2 calculations for **1**-**6** showing the equatorial manganese coordination core for the complexes (axial ligands and aromatic rings were hidden for clarity). The distances between the imine groups for complexes **3**-**6** are shorter leading to a pincer effect caused by the short aliphatic chain in contrast to the greater flexibility in the case of the longer aliphatic chain for complexes **1**-**2**

CheckCIF (a service of International Union of Crystallography) for **1**

CheckCIF (a service of International Union of Crystallography) for **3**

Table S1. Crystal data and refinement for **1** and **3**.

	**1**	**3**
Empirical formula	$C_{24}H_{32}MnN_3O_6S$	$C_{50}H_{78}Mn_2N_6O_{26}$
Formula weight	545.53	1289.06
Temperature [K]	100(2)	110(2)
Wavelength [Å]	0.71069	0.71069
Crystal system	Monoclinic	Triclinic
Space group	P 21/c	P-1
a [Å]	11.1715(7)	11.054(2)
b [Å]	15.6687(11)	11.829(2)
c [Å]	14.5784(9)	12.366(3)
α [°]	90	109.952(3)
β [°]	97.585(15)	95.283(3)
γ [°]	90	97.879(3)
Volume [$Å^3$]	2529.5(3)	1488.7
Z	4	1
Density (calculated) [g cm^{-3}]	1.432	1.438
Absorption coefficient [mm^{-1}]	0.649	0.512
Theta range for data collection [°]	1.84 to 26.02	1.77 to 26.02
Reflections collected	23347	16761
Independent reflections	4984	5855
Final R indices [I>2sigma(I)]	R1= 0.0308; $R_w(F^2)$ = 0.0687	R1= 0.0392; $R_w(F^2)$= 0.0949
R indices (all data)	R1= 0.0419; $R_w(F^2)$= 0.0725	R1= 0.0567; $R_w(F^2)$= 0.1040

Table S2. Selected bond lengths (A) and angles (º) for **1**.

Mn(1)-O(13a)	1.8844(12)	Mn(1)-O(13b)	1.8941(12)
Mn(1)-N(5a)	2.0372(15)	Mn(1)-N(5b)	2.0383(14)
Mn(1)-N(16)	2.1802(16)	Mn(1)-O(19)	2.2969(13)
O(13a)-C(12a)	1.321(2)	C(12b)-O(13b)	1.320(2)
C(6b)-N(5b)	1.288(2)	N(5a)-C(6a)	1.291(2)
C(4b)-N(5b)	1.475(2)	N(5a)-C(4a)	1.474(2)
N(16)-C(17)	1.160(2)	C(17)-S(18)	1.631(2)
O(14a)-C(11a)	1.369(2)		
C(20)-O(19)	1.442(2)		
O(13A)-Mn(1)-N(5A)	89.21(6)	O(13B)-Mn(1)-N(5B)	88.80(6)
O(13A)-Mn(1)-N(5B)	172.63(6)	O(13B)-Mn(1)-N(5A)	175.57(6)
O(13A)-Mn(1)-O(13B)	89.30(5)	N(5A)-Mn(1)-N(5B)	92.16(6)
O(13A)-Mn(1)-N(16)	95.58(6)	O(13B)-Mn(1)-N(16)	95.74(6)
N(5A)-Mn(1)-N(16)	88.55(6)	N(5B)-Mn(1)-N(16)	91.69(6)
O(13A)-Mn(1)-O(19)	87.04(5)	O(13B)-Mn(1)-O(19)	88.64(5)
N(5A)-Mn(1)-O(19)	87.12(5)	N(5B)-Mn(1)-O(19)	85.81(5)
N(16)-Mn(1)-O(19)	174.91(5)	N(16)-C(17)-S(18)	179.17(19)
C(12B)-O(13B)-Mn(1)	123.16(11)	C(12A)-O(13A)-Mn(1)	124.54(11)
C(4A)-N(5A)-Mn(1)	119.94(11)	C(4B)-N(5B)-Mn(1)	120.42(11)
C(6A)-N(5A)-Mn(1)	122.63(12)	C(6B)-N(5B)-Mn(1)	121.97(12)
C(4B)-C(2)-C(4A)	111.54(15)	C(1)-C(2)-C(3)	109.99(15)

Table S3. Selected bond lengths (Å) and angles (º) for **3**.

O(9)-Mn(1)	1.8779(15)	O(22)-Mn(1)	1.8819(16)
N(11)-Mn(1)	1.9695(19)	N(14)-Mn(1)	1.9800(19)
O(31)-Mn(1)	2.3072(17)	N(25)-Mn(1)	2.3296(19)
C(21)-O(22)	1.329(3)	C(8)-O(9)	1.322(3)
C(20)-O(23)	1.368(3)	O(2)-C(3)	1.364(3)
O(23)-C(24)	1.432(3)	C(1)-O(2)	1.427(3)
N(14)-C(15)	1.293(3)	C(10)-N(11)	1.290(3)
C(13)-N(14)	1.475(3)	N(11)-C(12)	1.476(3)
N(25)-C(26)	1.340(3)	N(25)-C(30)	1.343(3)
O(22)-Mn(1)-N(14)	92.45(7)	O(9)-Mn(1)-N(11)	91.03(7)
O(9)-Mn(1)-O(22)	94.14(7)	N(11)-Mn(1)-N(14)	82.40(8)
O(22)-Mn(1)-N(11)	174.79(7)	O(9)-Mn(1)-N(14)	173.29(7)
O(9)-Mn(1)-O(31)	91.21(7)	O(22)-Mn(1)-O(31)	90.11(7)
N(11)-Mn(1)-O(31)	89.09(7)	N(14)-Mn(1)-O(31)	90.05(7)
O(9)-Mn(1)-N(25)	89.69(7)	O(22)-Mn(1)-N(25)	88.71(7)
N(11)-Mn(1)-N(25)	92.02(7)	N(14)-Mn(1)-N(25)	89.19(7)
O(31)-Mn(1)-N(25)	178.56(7)	C(15)-N(14)-C(13)	119.0(2)
C(10)-N(11)-C(12)	121.6(2)	C(20)-O(23)-C(24)	117.54(18)
C(3)-O(2)-C(1)	117.30(19)	N(14)-C(15)-C(16)	125.3(2)
N(11)-C(10)-C(7)	124.7(2)	O(22)-C(21)-C(20)	117.3(2)
O(9)-C(8)-C(3)	117.4(2)		

Table S4. Hydrogen bond scheme (distances and angles) for **3**.

**D-H…A**	**d(D-H)**	**d(H…A)**	**d(D…A)**	**<(DHA)**
O(31)--H(31A)..O(22)[a]	0.78(4)	2.13(4)	2.838(3)	150(3)
O(31)--H(31A)..O(23)[a]	0.78(4)	2.26(4)	2.873(3)	136(3)
O(31) --H(31B) ..O(2)[a]	0.80(3)	2.23(3)	2.919(3)	145(3)
O(31) --H(31B) ..O(9)[a]	0.80(3)	2.19(3)	2.865(2)	142(3)'
O(36)--H(36A)..O(32)[b]	0.88(5)	1.91(5)	2.761(3)	163(4)
O(36)--H(36B)..O(33)[b]	0.87(4)	1.98(4)	2.799(3)	159(3)
O(37)--H(37A)..O(32)[c]	0.94(4)	1.87(4)	2.788(3)	166(3)
O(37)--H(37B)..O(38)[c]	0.82(5)	1.96(5)	2.774(3)	175(4)
O(38)--H(38A)..O(41)[d]	0.84(5)	1.92(5)	2.753(3)	168(4)
O(38)--H(38B)..O(40)[e]	0.85(6)	1.92(6)	2.762(3)	169(5)
O(39)--H(39A)..O(37)[e]	0.95(6)	1.82(6)	2.762(4)	177(5)
O(39)--H(39B)..O(31)[e]	0.88(3)	2.12(3)	2.988(3)	170(3)
O(40)--H(40A)..O(39)[e]	0.92(5)	1.81(6)	2.731(3)	178(5)
O(40)--H(40B)..O(33)[e]	0.85(3)	1.86(3)	2.710(3)	173(4)
O(41)--H(41A)..O(40)[e]	0.88(5)	1.96(5)	2.824(3)	165(4)
O(41)--H(41B)..O(36)[e]	0.89(3)	1.88(3)	2.753(3)	169(4)
Intra C(26)--H(26)…O(22)[h]	0.95	2.47	3.011(3)	116

Symmetry operations: [a] 1-x,1-y,-z; [b]-x,2-y,1-z; [c]-x,1-y,1-z; [d] 1-x,-y,1-z; [e] 1-x,1-y,1-z; [f] x,y,-1+z; [g] 1+x,y,z; [h] x,1+y,z

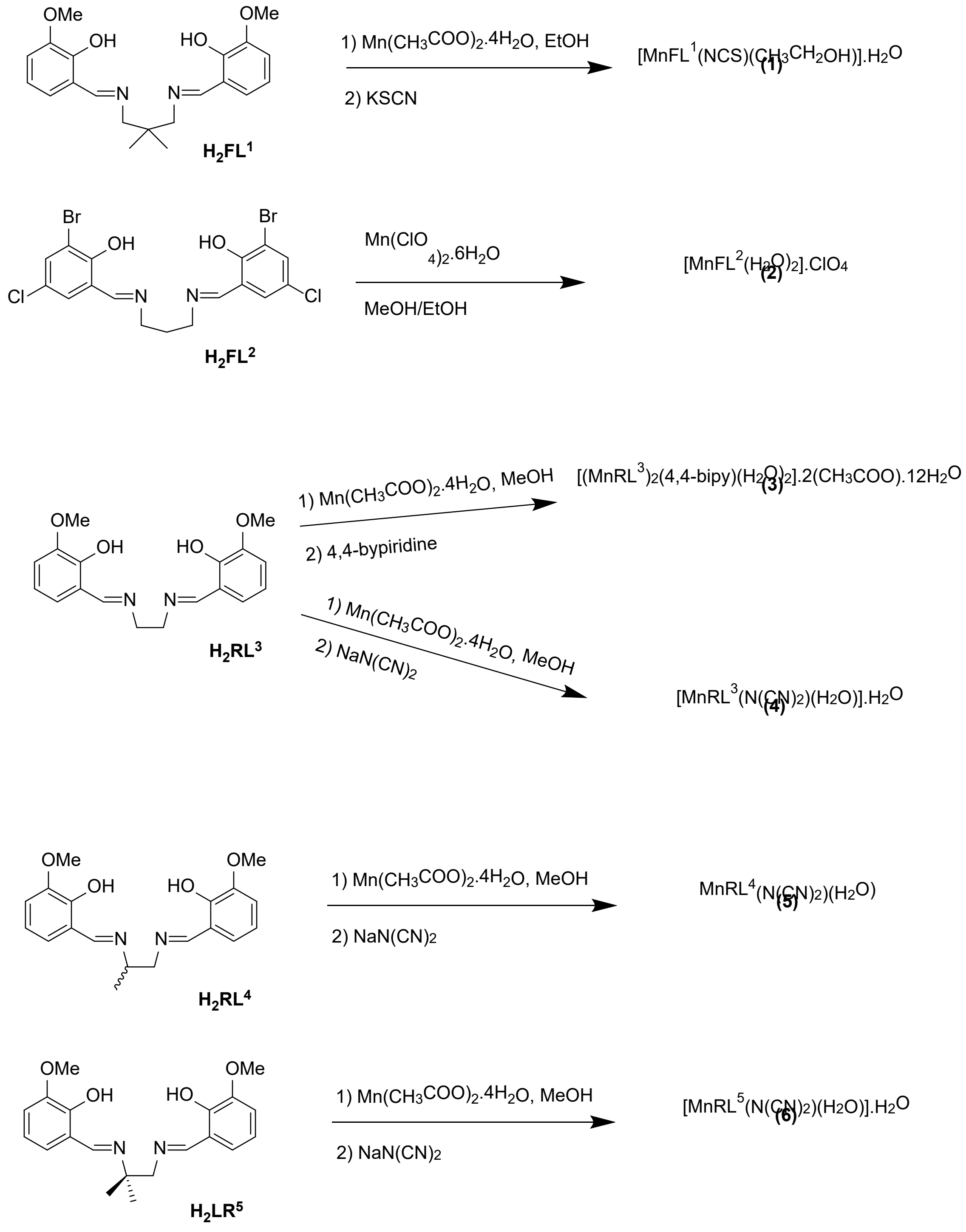

Scheme S1. Scheme of the Schiff base ligands H_2FL^1, H_2FL^2, H_2RL^3, H_2RL^4 and H_2RL^5, and their reactions to obtain complexes **1**-**6**. Synthesis and characterization for complexes **4**-**6** have been previously reported [39].

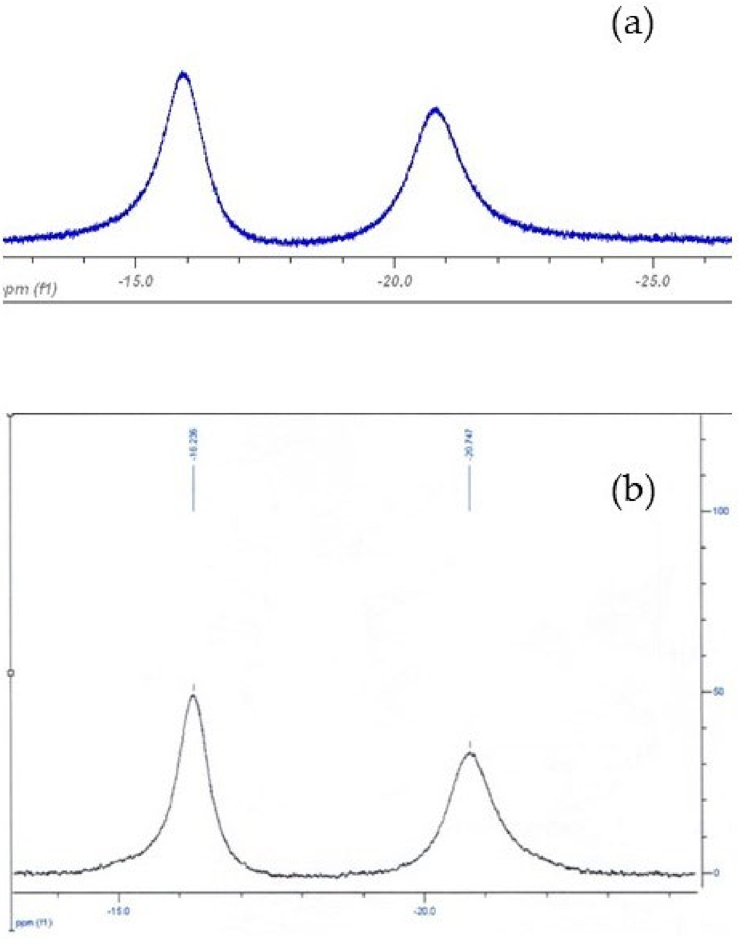

Figure S1. Paramagnetic ^{1}H NMR spectra for **1** (a) and **3** (b).

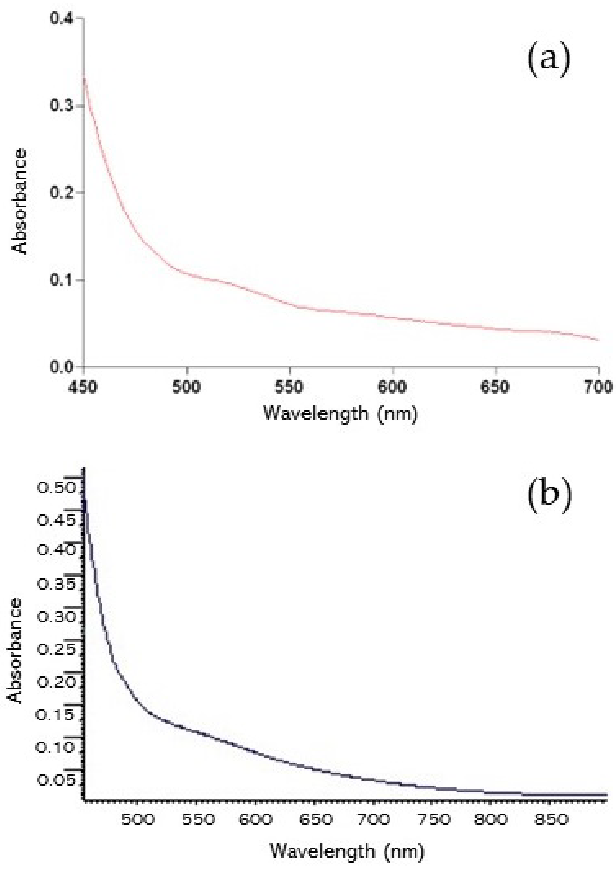

Figure S2. UV-vis spectra for **1** (a) and **3** (b).

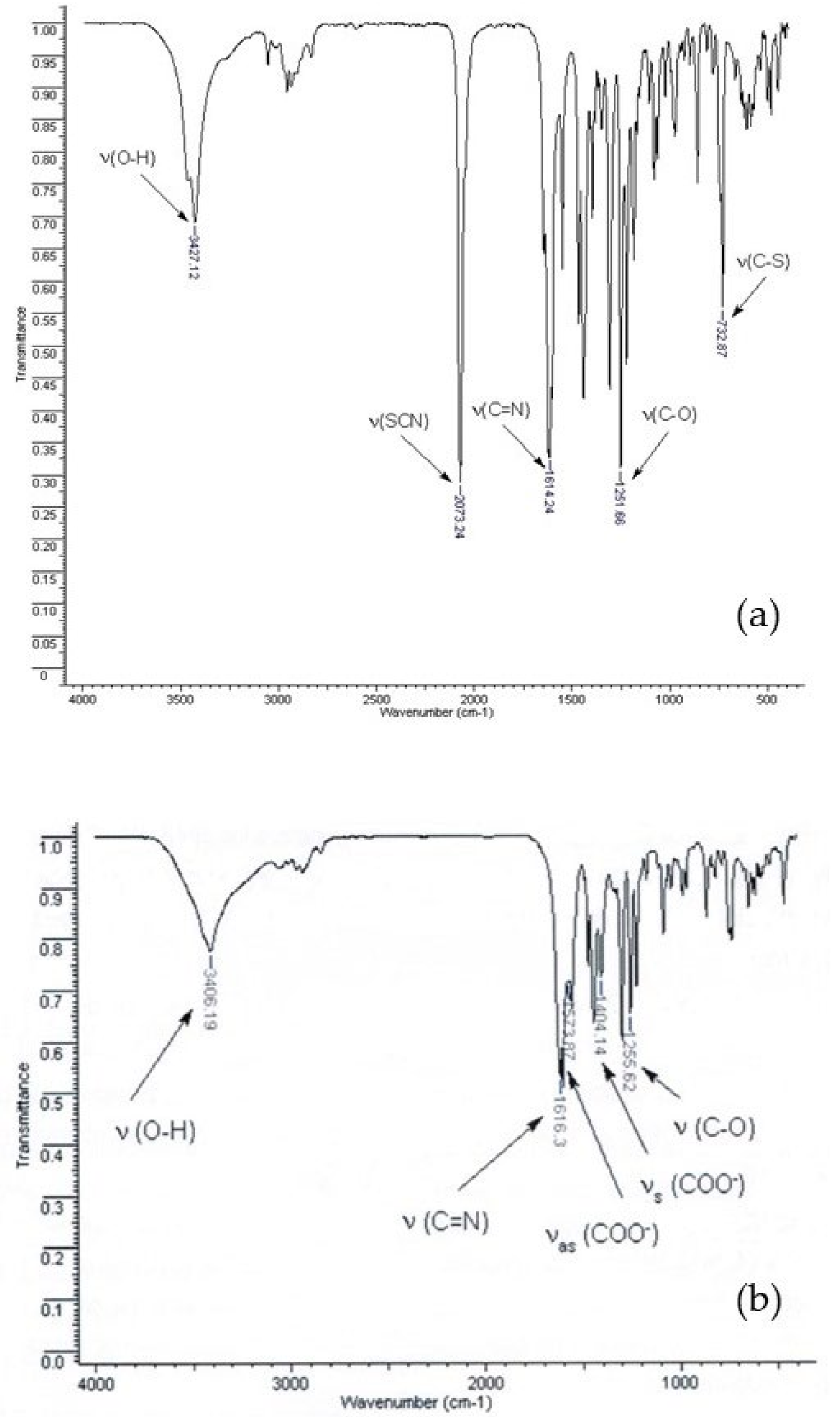

Figure S3. IR spectra for **1**(a) and **3** (b).

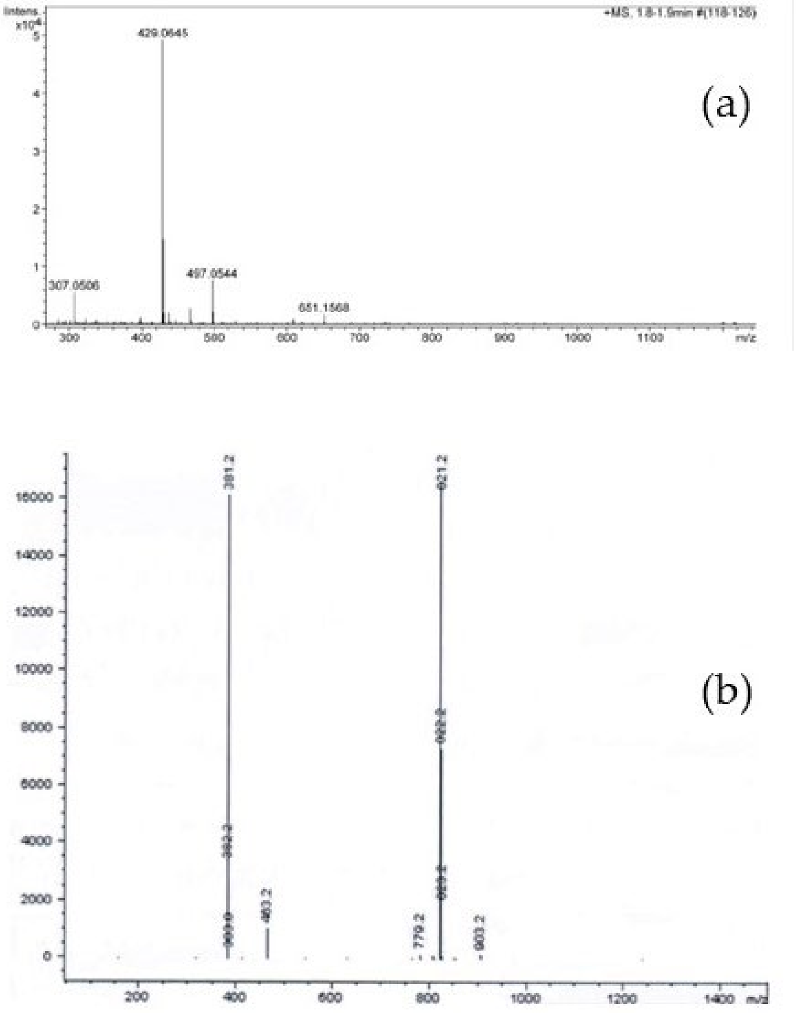

Figure S4. ESI mass spectra for **1** (a) and **3** (b).

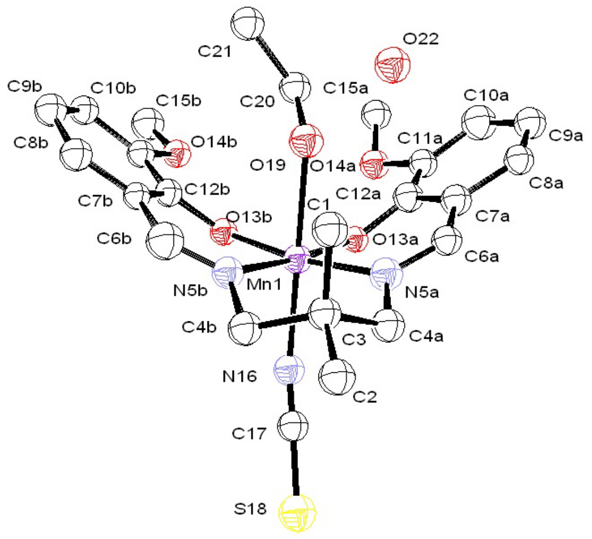

Figure S5. ORTEP view and atom numbering scheme for **1**, showing the coordination of the Schiff base ligand FL[1], the thiocyanate and ethanol molecule to the manganese ion; hydrogen atoms are omitted for clarity.

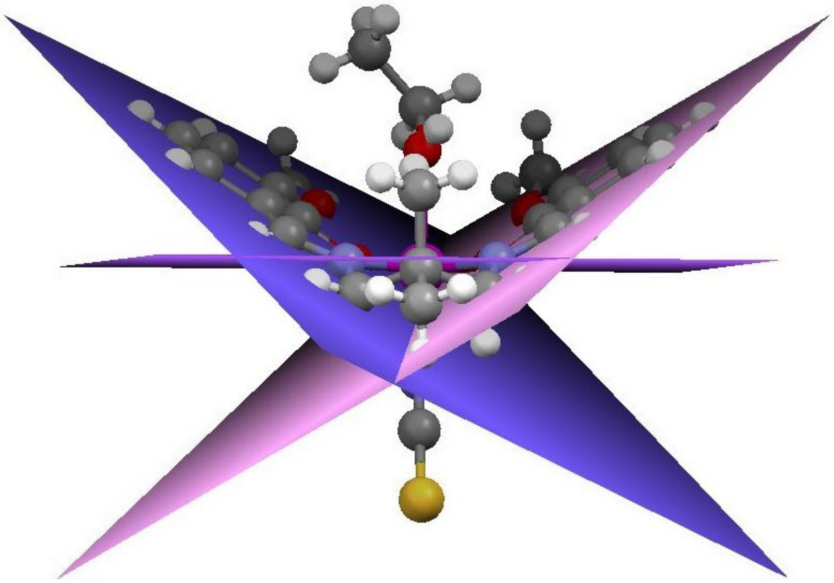

Figure S6. Representation of the dihedral angle (value of 66.74°) between the aromatic rings of the salen-type ligand in **1**.

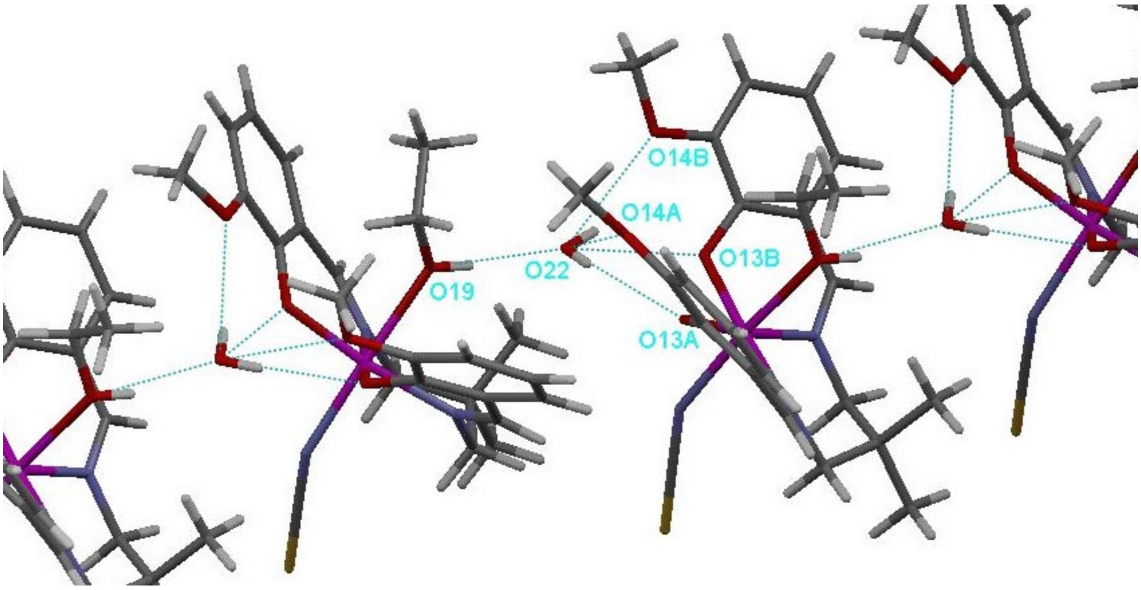

Figure S7. Stick diagram of **1** showing the supramolecular 1D chain through hydrogen bonding.

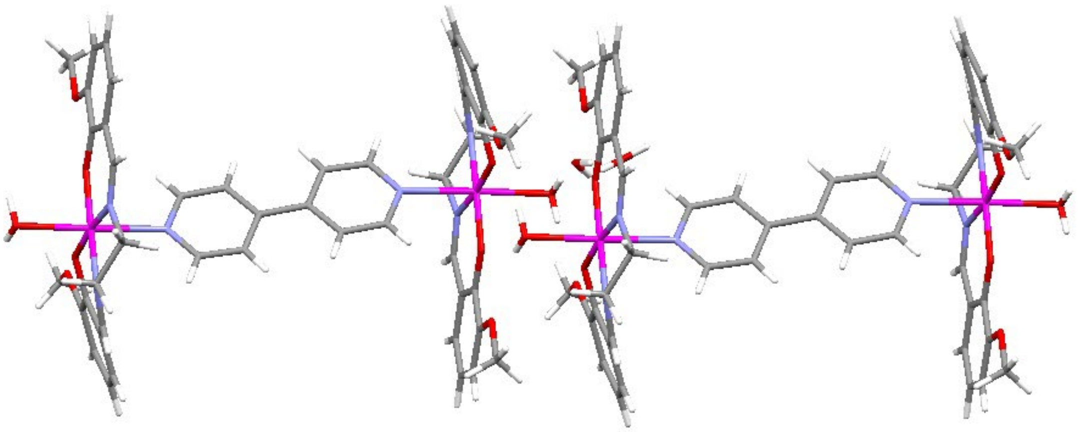

Figure S8. Hydrogen bonding between axial water molecules and iminic N atom and phenolic O atom from de neighboring dimeric complex in **3** causes intermolecular a Mn…Mn distance of 4.665 Å, shorter than the 11.710 Å for the Mn…Mn distance inside de dimer.

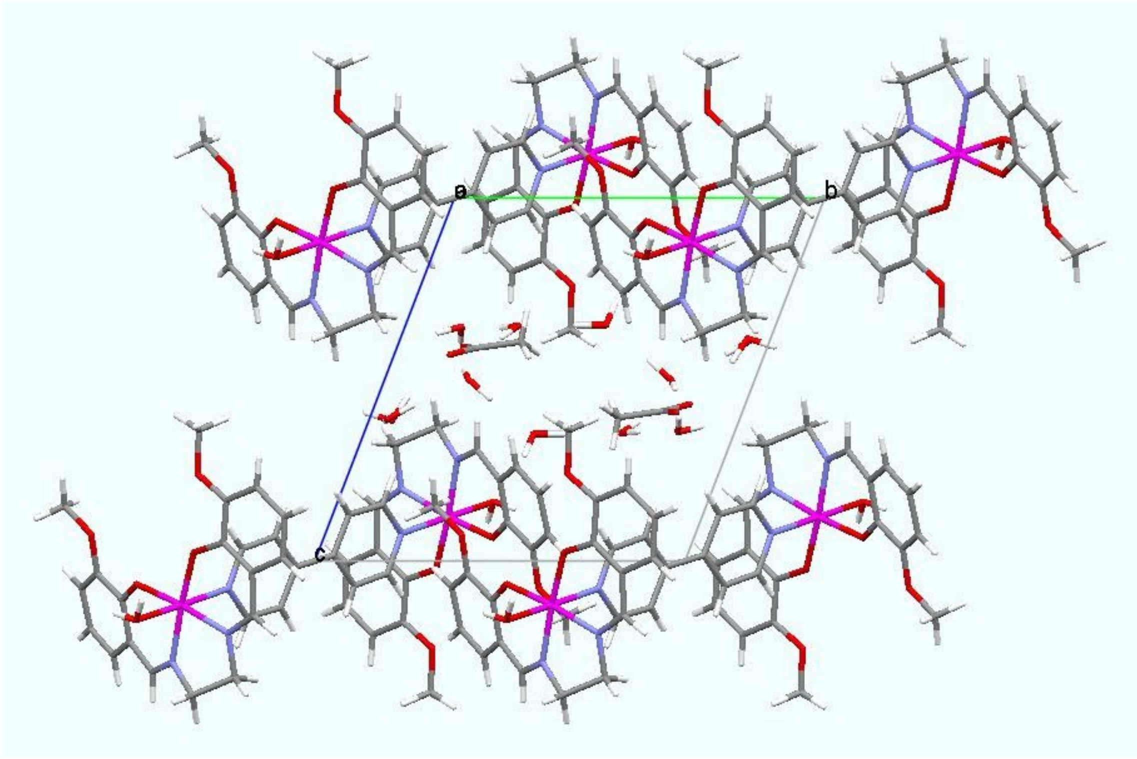

Figure S9. Three-dimensional a-axis view for **3**.

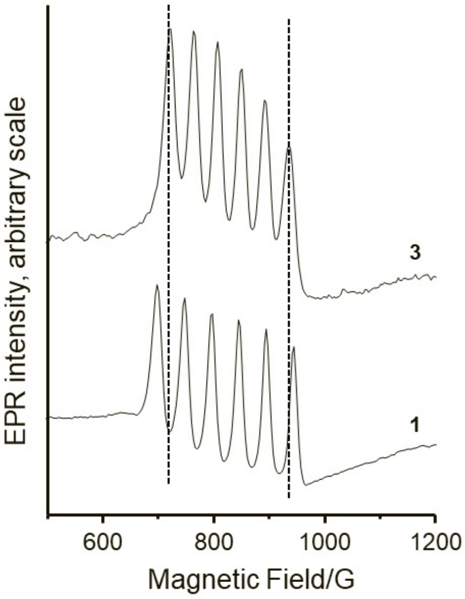

Figure S10. Parallel-mode electron paramagnetic resonance (EPR) spectra for **1** and **3** in frozen toluene:dimethylformamide:ethanol (2:1:drop) solutions. EPR conditions: microwave frequency, 9.37 GHz; temperature, 9 K; microwave power, 2 mW.

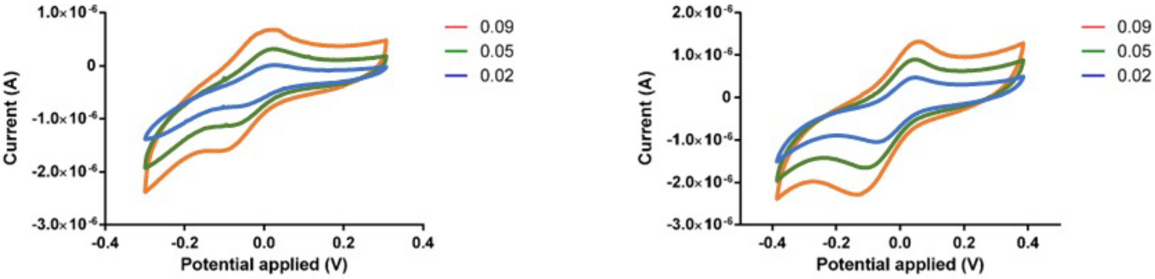

1 **3**

Figure S11. Cyclic voltammograms for **1** and **3** at different scan rates: 0.02 V s^{-1} (blue), 0.05 V s^{-1} (green) and 0.09 V s^{-1} (orange).

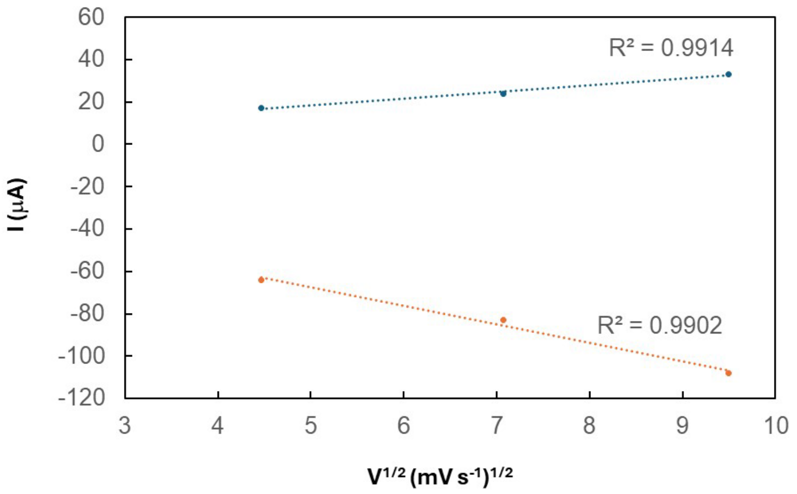

Figure S12. Plot of the linear dependence of anodic and cathodic peak currents with the square root of the scan rate of **1**.

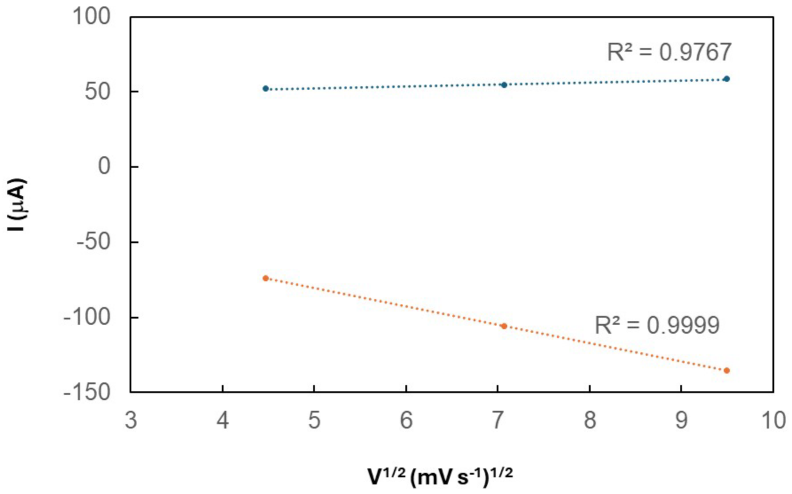

Figure S13. Plot of the linear dependence of anodic and cathodic peak currents with the square root of the scan rate of **3**.

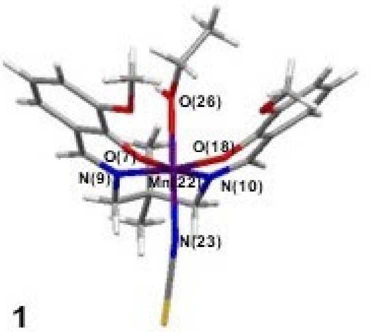

1

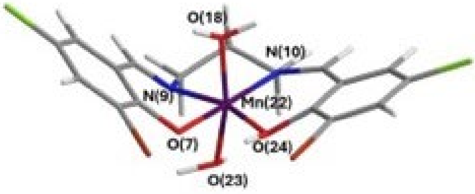

2

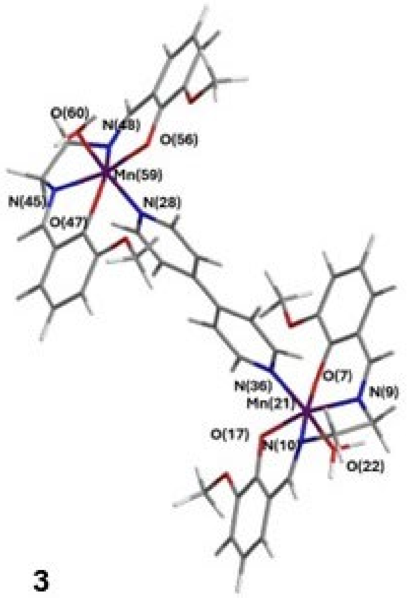

3

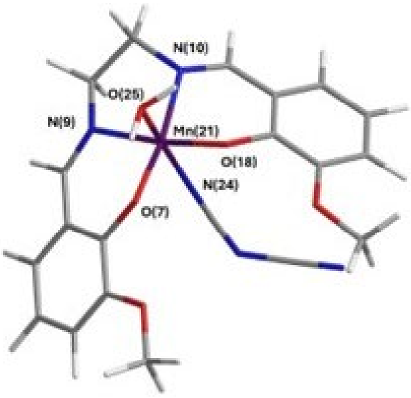

4

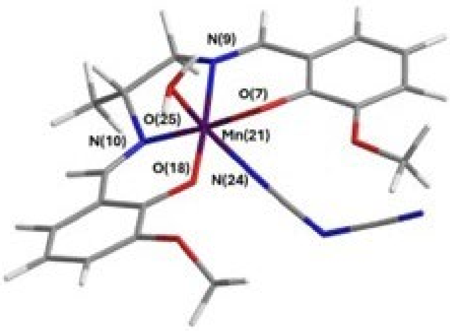

5

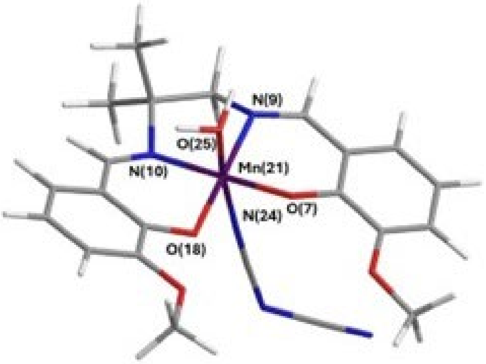

6

Figure S14. Simulation stick diagrams for **1**-**6** obtained by MM2 calculations to get the minimum energy perspective plots for the geometry of the complexes.

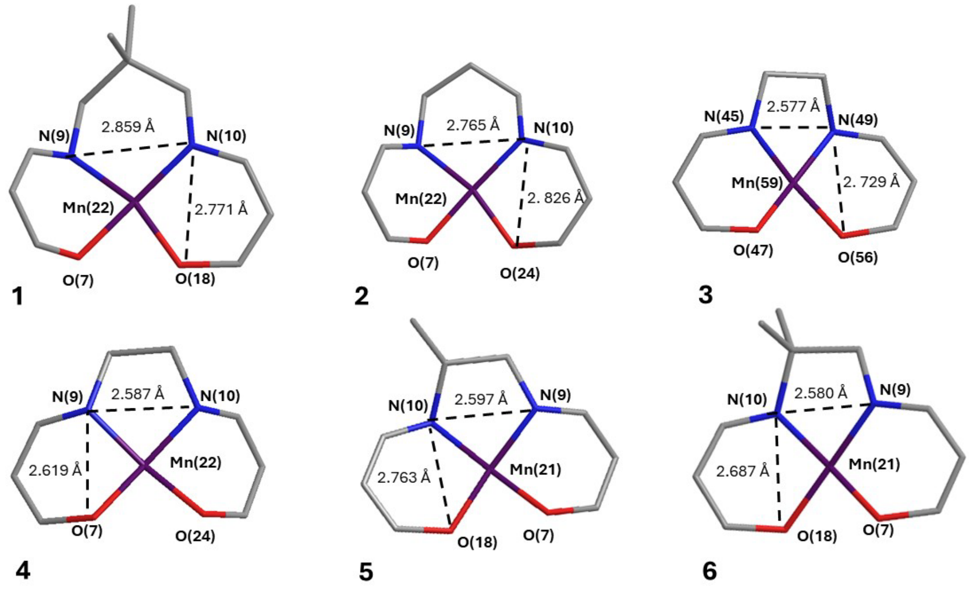

Figure S15. Stick diagram views obtained by MM2 calculations for **1**-**6** showing the equatorial manganese coordination core for the complexes (axial ligands and aromatic rings were hidden for clarity). The distances between the imine groups for complexes **3**-**6** are shorter leading to a pincer effect caused by the short aliphatic chain in contrast to the greater flexibility in the case of the longer aliphatic chain for complexes **1**-**2**.

checkCIF/PLATON report

You have not supplied any structure factors. As a result the full set of tests cannot be run.

THIS REPORT IS FOR GUIDANCE ONLY. IF USED AS PART OF A REVIEW PROCEDURE FOR PUBLICATION, IT SHOULD NOT REPLACE THE EXPERTISE OF AN EXPERIENCED CRYSTALLOGRAPHIC REFEREE.

No syntax errors found. CIF dictionary Interpreting this report

Datablock: complex1

```
Bond precision:   C-C = 0.0028 A          Wavelength=0.71073

Cell:             a=11.1715(7)     b=15.6687(11)      c=14.5784(9)
                  alpha=90         beta=97.585(3)     gamma=90
Temperature:      100 K

                 Calculated                  Reported
Volume           2529.5(3)                   2529.5(3)
Space group      P 21/c                      P 21/c
Hall group       -P 2ybc                     -P 2ybc
Moiety formula   C24 H30 Mn N3 O5 S, H2 O    C24 H30 Mn N3 O5 S, H2 O
Sum formula      C24 H32 Mn N3 O6 S          C24 H32 Mn N3 O6 S
Mr               545.53                      545.53
Dx,g cm-3        1.433                       1.432
Z                4                           4
Mu (mm-1)        0.649                       0.649
F000             1144.0                      1144.0
F000'            1146.30
h,k,lmax                                     13,19,17
Nref                                         4984
Tmin,Tmax        0.849,0.994                 0.906,1.000
Tmin'            0.802

Correction method= # Reported T Limits: Tmin=0.906 Tmax=1.000
AbsCorr = MULTI-SCAN

Data completeness=                 Theta(max)= 26.020

R(reflections)= 0.0308( 4079)                    wR2(reflections)= 0.0725( 4984)

S = 1.054                    Npar= 333
```

```
The following ALERTS were generated. Each ALERT has the format
       test-name_ALERT_alert-type_alert-level.
Click on the hyperlinks for more details of the test.
```

Alert level G

```
PLAT005_ALERT_5_G No Embedded Refinement Details Found  in the CIF     Please Do !
PLAT230_ALERT_2_G Hirshfeld Test Diff for    S18      --C17      .       10.7 s.u.
PLAT230_ALERT_2_G Hirshfeld Test Diff for    N16      --C17      .        5.5 s.u.
PLAT232_ALERT_2_G Hirshfeld Test Diff (M-X)  Mn1      --N5B      .        5.1 s.u.
PLAT794_ALERT_5_G Tentative Bond Valency for Mn1       (I)       .       0.82 Info
```

```
   0 ALERT level A = Most likely a serious problem - resolve or explain
   0 ALERT level B = A potentially serious problem, consider carefully
   0 ALERT level C = Check. Ensure it is not caused by an omission or oversight
   5 ALERT level G = General information/check it is not something unexpected

   0 ALERT type 1 CIF construction/syntax error, inconsistent or missing data
   3 ALERT type 2 Indicator that the structure model may be wrong or deficient
   0 ALERT type 3 Indicator that the structure quality may be low
   0 ALERT type 4 Improvement, methodology, query or suggestion
   2 ALERT type 5 Informative message, check
```

It is advisable to attempt to resolve as many as possible of the alerts in all categories. Often the minor alerts point to easily fixed oversights, errors and omissions in your CIF or refinement strategy, so attention to these fine details can be worthwhile. In order to resolve some of the more serious problems it may be necessary to carry out additional measurements or structure refinements. However, the purpose of your study may justify the reported deviations and the more serious of these should normally be commented upon in the discussion or experimental section of a paper or in the "special_details" fields of the CIF. checkCIF was carefully designed to identify outliers and unusual parameters, but every test has its limitations and alerts that are not important in a particular case may appear. Conversely, the absence of alerts does not guarantee there are no aspects of the results needing attention. It is up to the individual to critically assess their own results and, if necessary, seek expert advice.

Publication of your CIF in IUCr journals

A basic structural check has been run on your CIF. These basic checks will be run on all CIFs submitted for publication in IUCr journals (*Acta Crystallographica*, *Journal of Applied Crystallography*, *Journal of Synchrotron Radiation*); however, if you intend to submit to *Acta Crystallographica Section C* or *E* or *IUCrData*, you should make sure that full publication checks are run on the final version of your CIF prior to submission.

Publication of your CIF in other journals

Please refer to the *Notes for Authors* of the relevant journal for any special instructions relating to CIF submission.

PLATON version of 14/11/2023; check.def file version of 14/09/2023

Datablock complex1 - ellipsoid plot

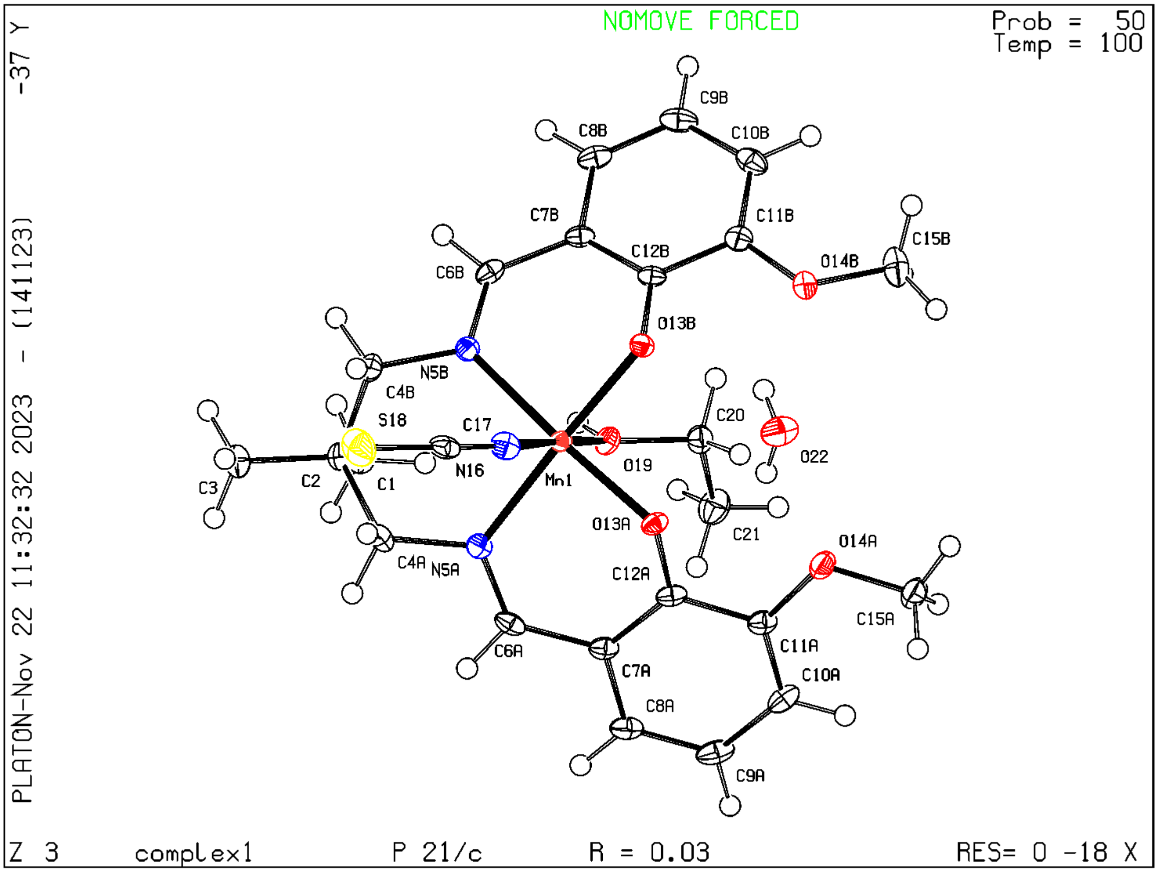

checkCIF/PLATON report

You have not supplied any structure factors. As a result the full set of tests cannot be run.

THIS REPORT IS FOR GUIDANCE ONLY. IF USED AS PART OF A REVIEW PROCEDURE FOR PUBLICATION, IT SHOULD NOT REPLACE THE EXPERTISE OF AN EXPERIENCED CRYSTALLOGRAPHIC REFEREE.

No syntax errors found. CIF dictionary Interpreting this report

Datablock: complex3

Bond precision: C-C = 0.0031 A Wavelength=0.71069

Cell:	a=11.054(2)	b=11.829(2)	c=12.366(3)
	alpha=109.952(3)	beta=95.283(3)	gamma=97.879(3)
Temperature:	110 K		

	Calculated	Reported
Volume	1488.7(5)	1488.7(5)
Space group	P -1	P -1
Hall group	-P 1	?
Moiety formula	C46 H48 Mn2 N6 O10, 2(C2 H3 O2), 12(H2 O)	C46 H48 Mn2 N6 O10, 2(C2 H3 O2), 12(H2 O)
Sum formula	C50 H78 Mn2 N6 O26	C25 H39 Mn N3 O13
Mr	1289.06	644.53
Dx,g cm-3	1.438	1.438
Z	1	2
Mu (mm-1)	0.512	0.512
F000	678.0	678.0
F000'	679.05	
h,k,lmax		13,14,15
Nref		5855
Tmin,Tmax	0.858,0.926	0.849,0.927
Tmin'	0.845	

Correction method= # Reported T Limits: Tmin=0.849 Tmax=0.927
AbsCorr = MULTI-SCAN

Data completeness= Theta(max)= 26.020

R(reflections)= 0.0392(4649) wR2(reflections)= 0.1040(5855)

S = 1.051 Npar= 438

The following ALERTS were generated. Each ALERT has the format
test-name_ALERT_alert-type_alert-level.
Click on the hyperlinks for more details of the test.

Alert level C

```
PLAT314_ALERT_2_C Small Angle for H2O: Metal-O31      -H31A      .      94.59 Degree
```

Alert level G

```
FORMU01_ALERT_1_G  There is a discrepancy between the atom counts in the
            _chemical_formula_sum and _chemical_formula_moiety. This is
            usually due to the moiety formula being in the wrong format.
            Atom count from _chemical_formula_sum:   C25 H39 Mn1 N3 O13
            Atom count from _chemical_formula_moiety:C50 H78 Mn2 N6 O26
PLAT005_ALERT_5_G No Embedded Refinement Details Found  in the CIF     Please Do !
PLAT045_ALERT_1_G Calculated and Reported Z Differ by a Factor ...      0.500 Check
PLAT066_ALERT_1_G Predicted and Reported Tmin&Tmax Range Identical          ? Check
PLAT154_ALERT_1_G The s.u.'s on the Cell Angles are Equal ..(Note)      0.003 Degree
PLAT710_ALERT_4_G Delete 1-2-3 or 2-3-4 Linear Torsion Angle ... #         62 Do !
              C8  -O9  -MN1 -N14    14.70  0.70   1_555   1_555   1_555   1_555
PLAT710_ALERT_4_G Delete 1-2-3 or 2-3-4 Linear Torsion Angle ... #         66 Do !
              C21 -O22 -MN1 -N11    -7.30  0.90   1_555   1_555   1_555   1_555
PLAT710_ALERT_4_G Delete 1-2-3 or 2-3-4 Linear Torsion Angle ... #         72 Do !
              C10 -N11 -MN1 -O22   167.40  0.70   1_555   1_555   1_555   1_555
PLAT710_ALERT_4_G Delete 1-2-3 or 2-3-4 Linear Torsion Angle ... #         73 Do !
              C12 -N11 -MN1 -O22   -10.10  0.90   1_555   1_555   1_555   1_555
PLAT710_ALERT_4_G Delete 1-2-3 or 2-3-4 Linear Torsion Angle ... #         80 Do !
              C15 -N14 -MN1 -O9   -170.80  0.60   1_555   1_555   1_555   1_555
PLAT710_ALERT_4_G Delete 1-2-3 or 2-3-4 Linear Torsion Angle ... #         81 Do !
              C13 -N14 -MN1 -O9      5.50  0.70   1_555   1_555   1_555   1_555
PLAT710_ALERT_4_G Delete 1-2-3 or 2-3-4 Linear Torsion Angle ... #         98 Do !
              C26 -N25 -MN1 -O31    46.00  3.00   1_555   1_555   1_555   1_555
PLAT710_ALERT_4_G Delete 1-2-3 or 2-3-4 Linear Torsion Angle ... #         99 Do !
              C30 -N25 -MN1 -O31  -123.00  3.00   1_555   1_555   1_555   1_555
PLAT794_ALERT_5_G Tentative Bond Valency for Mn1       (I)       .       0.84 Info
PLAT881_ALERT_1_G No Datum for _diffrn_reflns_av_R_equivalents ...     Please Do !
PLAT899_ALERT_4_G SHELXL-97  is Deprecated and Succeeded by SHELXL     2019/3 Note
```

0 **ALERT level A** = Most likely a serious problem - resolve or explain
0 **ALERT level B** = A potentially serious problem, consider carefully
1 **ALERT level C** = Check. Ensure it is not caused by an omission or oversight
16 **ALERT level G** = General information/check it is not something unexpected

5 ALERT type 1 CIF construction/syntax error, inconsistent or missing data
1 ALERT type 2 Indicator that the structure model may be wrong or deficient
0 ALERT type 3 Indicator that the structure quality may be low
9 ALERT type 4 Improvement, methodology, query or suggestion
2 ALERT type 5 Informative message, check

It is advisable to attempt to resolve as many as possible of the alerts in all categories. Often the minor alerts point to easily fixed oversights, errors and omissions in your CIF or refinement strategy, so attention to these fine details can be worthwhile. In order to resolve some of the more serious problems it may be necessary to carry out additional measurements or structure refinements. However, the purpose of your study may justify the reported deviations and the more serious of these should normally be commented upon in the discussion or experimental section of a paper or in the "special_details" fields of the CIF. checkCIF was carefully designed to identify outliers and unusual parameters, but every test has its limitations and alerts that are not important in a particular case may appear. Conversely, the absence of alerts does not guarantee there are no aspects of the results needing attention. It is up to the individual to critically assess their own results and, if necessary, seek expert advice.

Publication of your CIF in IUCr journals

A basic structural check has been run on your CIF. These basic checks will be run on all CIFs submitted for publication in IUCr journals (*Acta Crystallographica*, *Journal of Applied Crystallography*, *Journal of Synchrotron Radiation*); however, if you intend to submit to *Acta Crystallographica Section C* or *E* or *IUCrData*, you should make sure that full publication checks are run on the final version of your CIF prior to submission.

Publication of your CIF in other journals

Please refer to the *Notes for Authors* of the relevant journal for any special instructions relating to CIF submission.

PLATON version of 14/11/2023; check.def file version of 14/09/2023

Datablock complex3 - ellipsoid plot

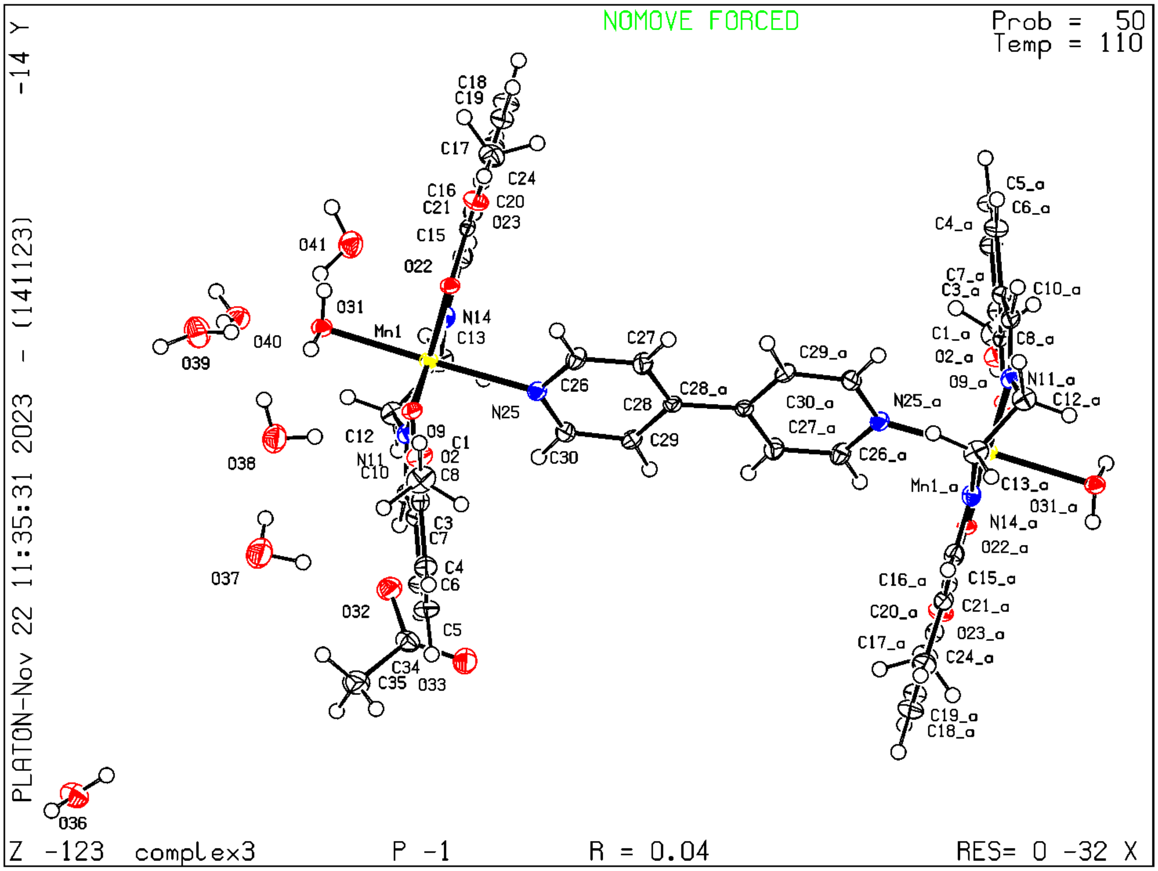

Supplement: Supplementary file 1 [file antioxidants-13-00265-s001.zip › antioxidants-2864810-supplementary.pdf]
